# Improving Protein Structure Determination by Integrating Ensemble-Driven Molecular Dynamics with Chemical Shift-Based Restraints

**DOI:** 10.1021/acs.jcim.5c02358

**Published:** 2026-02-16

**Authors:** Márton Gadanecz, Zsolt Fazekas, Dóra K. Menyhárd, András Perczel

**Affiliations:** † Laboratory of Structural Chemistry and Biology, Institute of Chemistry, 431371 Eötvös Loránd University, Pázmány Péter Stny. 1/A, Budapest H-1117, Hungary; ‡ Hevesy György PhD School of Chemistry, Eötvös Loránd University, Pázmány Péter Stny. 1/A, Budapest H-1117, Hungary; § HUN-REN−ELTE Protein Modelling Research Group, Eötvös Loránd Research Network (ELKH), Pázmány Péter Stny. 1/A, Budapest H-1117, Hungary

## Abstract

We present a protocol for nuclear magnetic resonance (NMR) chemical shift-based structure determination that employs ensemble-driven molecular dynamics (EDMD) for structure refinement. Here, specifically, Chemical-Shift-Rosetta (CS-Rosetta) was applied, followed by EDMD. EDMD eliminates the need to predict chemical shifts at every molecular dynamics (MD) step by defining continuous, differentiable potential energy functions (PEFs) based on dihedral angle distributions from CS-Rosetta models while incorporating the measurement temperature. This yielded a thermodynamically realistic, experiment-based custom force field for each studied system. We benchmarked EDMD against 5 proteins (13.1–19.2 kDa), focusing on systems with nonprotein components and demonstrated its consistent improvement of backbone root-mean-square deviation (RMSD) relative to known reference structures over the original CS-Rosetta ensemble. Moreover, EDMD enhanced the fulfillment of NOE-derived (nuclear Overhauser effect) distance restraints compared to the results of CS-Rosetta and unrestrained MD simulations. EDMD also improved NOE-RASREC-Rosetta (resolution-adapted structural recombination Rosetta protocol supplemented with NOE-based distance restraints) models and maintained the correct protein–ligand conformations. This approach provides an opportunity to refine nonconverged CS-Rosetta structure calculations, where the results would not be interpretable otherwise. EDMD can be generalized to any ensemble with scoring information, enabling refined exploration of the φ/ψ phase space and accurate reinsertion of nonprotein moieties.

## Graphical abstract

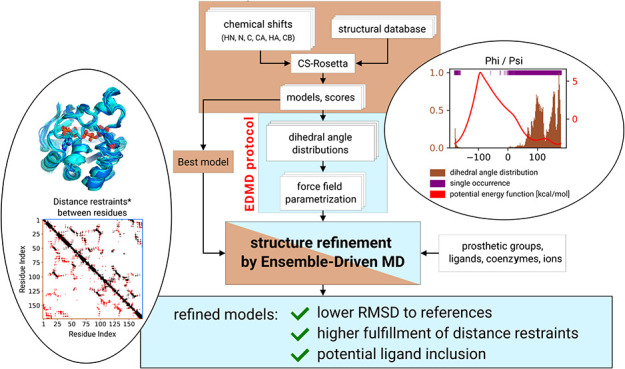

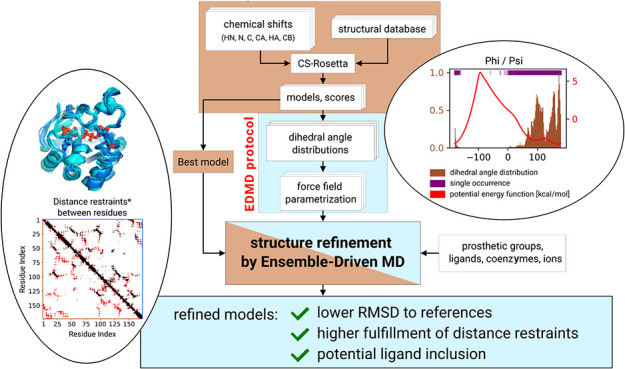

## 1 Introduction

NMR spectroscopy remains one of the most powerful and versatile techniques for studying biomacromolecules in solution. The information gained is multilayered and alludes to overall movement,^1,2^ flexibility,^3–5^ accessibility,^6^ and interconnectedness^7,8^ of the studied systems, be it *in vitro* or even in a cellular environment.^5,9^ Building 3D structures may still be challenging due to broadened or overlapping signals, particularly in flexible regions. However, even the most basic NMR observable, the chemical shift of a characteristic atom, carries nuanced structural information and is nearly always obtainable, making it a robust measurement. Thus, it is unsurprising that significant efforts are being made to develop reliable methodologies that can calculate 3D structures primarily on the basis of chemical shift data.

For protein structure determination, the most popular approach is the torsion angle dynamics method (e.g., CYANA^10^), which primarily relies on distance restraints derived from NOE^7^ (nuclear Overhauser effect) measurements, in addition to chemical shift-based backbone dihedral angle restraints. Recently, the process of peak picking, signal assignment, structural restraint definition, and CYANA-type structure calculation was automatized in the framework of ARTINA.^11^ Using ARTINA significantly reduces the manual input required for the otherwise time-consuming process of NMR protein structure elucidation. One disadvantage of this classical structure determination method is its high dependency on NOEs. The measurement of NOESY-type (nuclear Overhauser effect spectroscopy^7^) spectra takes a relatively long time. Moreover, similar TOCSY-type (total correlation spectroscopy^12^) data must also be acquired to assign the chemical shifts of side chain atoms. In addition to the long instrument time, atoms in flexible regions or regions surrounded by solvent molecules often do not produce valuable long-range NOE signals because of signal broadening, spin diffusion, and the lack of other nearby regions, respectively.

To avoid the problems associated with defining NOE-derived distance restraints, new strategies were developed relying on other NMR observables, such as residual dipolar couplings (RDCs) and chemical shifts.^13^ The relationship between NOEs and interatomic distances, or RDCs and pairwise orientation, can easily be described with a single equation. However, although the correlation between backbone chemical shifts and protein structure has been established for decades,^14^ their connection is more complicated. Chemical shift-based structure determination methods, like Cheshire,^15^ CS23D,^16^ or Chemical-Shift-Rosetta^17,18^ (CS-Rosetta), utilize chemical shift data through a comparison against back-calculated values of known structures.^19^ The collected high-resolution backbone fragments, selected based on sequence and chemical shift conjunctions, are sampled by a Monte Carlo (MC) algorithm. While the huge number of MC steps during structure determination provides robustness, this approach suffers from the majority of the trial moves being rejected. In addition to the inefficiency of the trial-and-error method, the chemical shift scoring terms derived from SPARTA/SPARTA+^20^ or SHIFTX/SHIFTX2 ^21^ calculations are noncontinuous, therefore nondifferentiable. Consequently, the restraints derived from chemical shifts cannot be directly used to uniformly explore the φ/ψ phase space.

Based on the number of entries in the Protein Data Bank (PDB) (as of July 2025), the most popular chemical shift-based structure determination software is CS-Rosetta. In this context, two CS-Rosetta structure calculation protocols are worth mentioning: the Abrelax (ab initio relax) and the RASREC (resolution-adapted structural recombination) schemes. All CS-Rosetta procedures use predicted chemical shift data in conjunction with the corresponding high-resolution crystal structures in a database. The procedures walk along the sequence of the queried protein using a sliding window of a few residue lengths and identify sequentially similar segments in the crystal structures of the database where the predicted chemical shift of the participating atoms has been found similar to that measured for the query protein. After this fragment-picking step, the classical CS-Rosetta protocol—Abrelax—calculates independent folding trajectories by assembling fragments and refining the candidate structures utilizing Rosetta’s Metropolis MC procedure. This protocol is also available on the web server of BMRB (https://csrosetta.bmrb.io). RASREC is a new protocol developed to address conformational sampling problems in proteins containing complex topologies. The RASREC-Rosetta is a multistage, iterative approach: at the early stages, it identifies low-energy models, which are iteratively resampled and recombined in later stages to progressively improve the models while maintaining diversity.^18,19^

Robustelli et al.^22^ introduced an *ab inito* structure determination method for small proteins. In this method, the protein chain folds from an extended conformation using chemical shifts as structural restraints, without the use of a homology database or molecular fragment libraries. They utilized SHIFTX predictions with MC rearrangements and an extra term in the force field to penalize chemical shift discrepancies.^22^ However, the method performs less successfully if the starting structure is far from the target structure: the sampling of the conformational space (and the convergence of the calculation) is time-consuming, as small structural changes can lead to large changes in the predicted chemical shifts and large energetic penalties. Since the discontinuous chemical shift predicting functions of SHIFTX require the use of an MC algorithm, the large number of rejected MC moves remains a challenge. This issue can be circumvented by using MD simulations for chemical shift-based structure building. ChemShift,^23^ one such protocol, predicts chemical shifts from polynomial functions of interatomic distances. These functions are differentiable with respect to the atomic coordinates; therefore, MC sampling can be replaced by MD simulations to explore the conformational space. As all-atom MD simulations are not a very effective way to fold proteins from extended conformations, Robustelli et al.^24^ suggested utilizing MD with chemical shift-based restraints as a refinement method for structure candidates provided by molecular fragment replacement techniques, like Cheshire, CS23D, or CS-Rosetta. In the work of Leelananda S.P. and Lindert S.,^25^ Rosetta models were generated (without any experimental data) and refined by an iterative protocol incorporating short MDFF (MD with flexible fitting^26^) simulations and Rosetta calculations utilizing chemical shift-based fragments. Cryo-EM density maps and backbone chemical shift information (using PLUMED^27^) were applied during the MDFF steps.

We have recently published a methodology^28^ that uses a novel approach to obtain conformational ensembles reflecting the measured chemical shifts, based on the CS-Rosetta *ab initio* structure determination protocol, while also providing the means for integrating nonprotein components (that are not considered by CS-Rosetta) into the process. The most direct link between the NMR measurements and the CS-Rosetta structure elucidation process is the φ (phi angle: C_i–1_–N_i_–C_αi_–C_i_) and ψ (psi angle: N_i_–C_αi_–C_i_–N_i+1_) torsion angle distributions of the model ensemble; therefore, we focused our protocol on these values. Kernel density estimation (KDE) and Boltzmann inversion were applied to define a potential energy function (PEF) for each backbone torsion angle—based on the values it samples in the ensembles generated by CS-Rosetta (weighting the models by the Rosetta score). These PEFs were then used to define restraining forces on backbone φ- and ψ-torsions, replacing the original AMBER99 torsional forces, in a chemical-shift-driven molecular dynamics (csdMD) simulation. By defining continuous and differentiable PEFs, it is possible to perform uniform exploration of the phase space around the local minima, resulting in refined, better-quality models, but without the need to calculate chemical shifts after every step of the MD simulation.

Here, we present a benchmarking of a refined version of this protocol, where the temperature was introduced during the PEF definition to provide robustness, with a special focus on the inclusion of prosthetic groups and other nonprotein components into the final structure. We have selected 5 different protein structures for which either or both NMR-based and crystallographically determined structures are available in the Protein Data Bank (PDB), and their measured chemical shift data are also retrievable from Biological Magnetic Resonance Bank, namely: cellular retinol-binding protein (CRBP)^29,30^ in complex with its cargo, the fluorescent flavin mononucleotide-binding protein (FBP)^31,32^ in complex with flavin, the GDP-bound resting state of a major human oncoprotein KRAS,^33–35^ the regulator of Ty1 transposition protein (RTT103),^36,37^ and the NADP­(H)-binding component (domain III) of proton-translocating transhydrogenase (THdIII).^38,39^ The selected proteins differ in their protein fold type, covering all the globular CATH^40^ classes (derived from the gross secondary structure content). RTT103 consists exclusively of alpha-helices. The structures of CRBP and FBP belong to the mainly beta class, as they are both beta-barrels with some alpha-helices on the periphery. KRAS and THdIII classify as as alpha-beta folds. The reference structures are shown in Figure 1A. Since CS-Rosetta was designed to solve the structure of globular proteins, we have not included intrinsically disordered proteins in this analysis. Four of the 5 selected systems contain some kind of nonprotein component(s).

**Figure 1:**
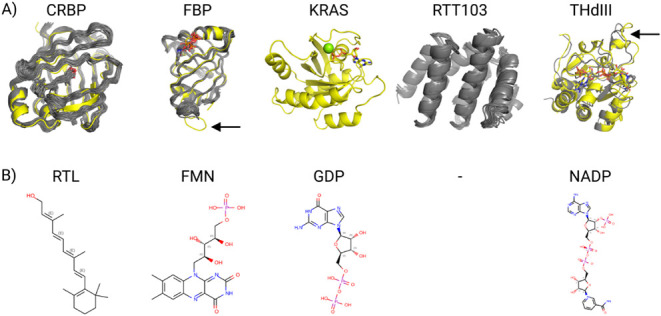
A) An overview of the superimposed reference X-ray crystal structures (yellow) and NMR structures (gray). Cellular Retinol-Binding Protein (CRBP),^29,30^ Flavin Mononucleotide-Binding Protein (FBP),^31,32^ GDP-bound state of wild-type Kirsten Rat Sarcoma viral oncogene homologue KRAS,^33–35^ Regulator of Ty1 Transposition protein (RTT103),^36,37^ and NADP­(H) binding component (domain III) of Proton-Translocating Transhydrogenase (THdIII).^38,39^ The protein backbones are depicted as cartoons, the small molecules as sticks, and the Mg^2+^ in KRAS as a sphere. The reference X-ray crystal structures and NMR structures from the Protein Data Bank (PDB) are 5LJB and 1MX8 for CRBP, chain A of 1FLM and 1AXJ for FBP, chain A of 4OBE for KRAS, 5WOZ for RTT103, and chain B of 1PNO and 1E3T for THdIII. B) The nonprotein component(s) of the systems are shown as sticks. There is retinol (Ligand ID: RTL) in CRBP, flavin mononucleotide (riboflavin-5′-phosphate, Ligand ID: FMN) in FBP, guanosine diphosphate (Ligand ID: GDP) plus a Mg^2+^ ion in KRAS, and nicotinamide adenine dinucleotide phosphate (NADP, Ligand ID: NAP) in THdIII. Differences between the reference structures are labeled with black arrows.

## 2 Results

### 2.1 *Ab Initio* Structure Generation by CS-Rosetta

We used CS-Rosetta to calculate model ensembles for each of the 5 systems in question based on chemical shift data retrieved from the Biological Magnetic Resonance Bank (BMRB). We did not supplement the calculations with NOE-based distance restraints or any other structural information. In the first stage, CS-Rosetta collected 3- and 9-residue-long fragments from the database based on backbone chemical shift and sequence similarity. In the second phase, 10,000 models were generated using the Abrelax protocol. One way to decide if a structure calculation has reached a robust end point is to check the root-mean-square deviation (RMSD) of the best-scoring models with respect to the overall best model. According to the CS-Rosetta software, 3 out of 5 structure determination processes carried out in this study (those of CRBP, FBP, and KRAS) have converged (Figure 2A). The average backbone RMSD and its standard deviations (see Figure 4 and Tables S1 and S2) also illustrate the same results. However, we found that even in the case of nonconvergent systems, the core structures of the best-scoring models align sufficiently well (see Figure 2B) to warrant the continuation of the structure calculation process.

**Figure 2:**
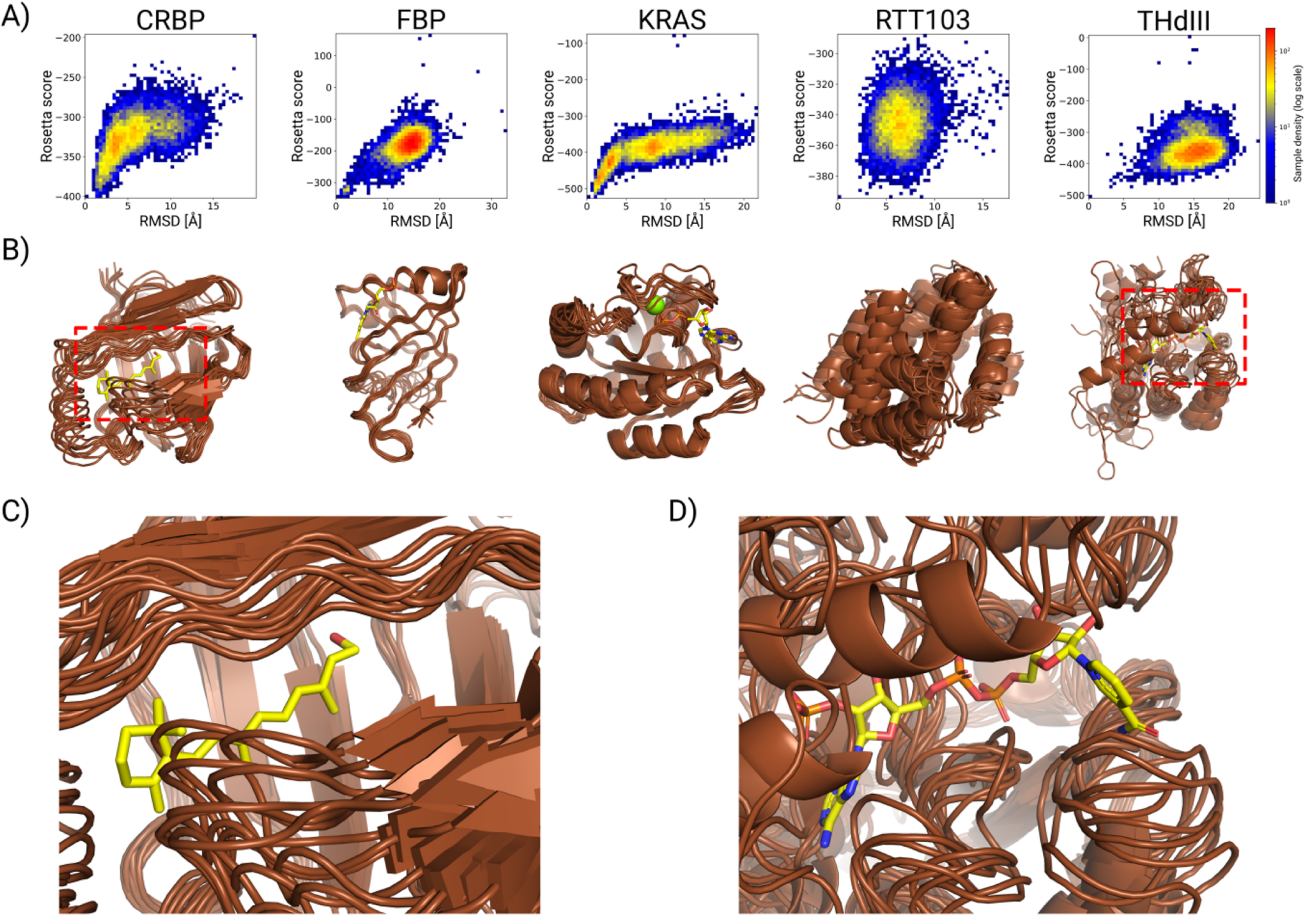
A) 2D histograms of Rosetta scores vs backbone RMSD relative to the best-scoring model. B) The superimposed best 10 models of the CS-Rosetta Abrelax structure calculations in the case of the systems under investigation. The protein backbones from the CS-Rosetta calculations are shown as brown cartoons, while the missing nonprotein components from the crystal structures are represented as red sticks. C) The retinol-binding pocket of CRBP (brown) with the missing RTL (yellow) from the experimental X-ray model. D) The binding NADP pocket of THdIII (brown) with the missing NADP (yellow) from the experimental X-ray structure.

In Figure 2C and D, the binding pockets of CRBP and THdIII are depicted to highlight the problem of creating structural models in the absence of the partner molecules (RTL and NADP in the case of CRBP and THdIII, respectively). Even among the top 10 models, in some models, the protein segments overlap with the binding site of the ligands, despite those components being present during the NMR data acquisition. In our belief, these models cannot be considered as final results; the small molecules must be reinserted, and the models must be accordingly refined.

### 2.2 Structure Refinement by Ensemble-Driven Molecular Dynamics

To refine the CS-Rosetta ensemble, we have applied a procedure we will refer to as Ensemble-driven MD (EDMD)—an improved version of our recently published chemical-shift-driven MD (csdMD) method.^28^ In this protocol, the entire CS-Rosetta ensemble and the corresponding Rosetta scores were used to define the weighted PEFs. As shown in Figure 3B, the PEF has the lowest value where the given dihedral angle is most likely to occur in the CS-Rosetta structure ensemble. In the csdMD method, the standard deviations of the individual PEFs were scaled one by one to the typical standard deviation of the torsional forces in the original force field, which resulted in different effective temperatures in the case of every modified dihedral angle. This also made it possible to emphasize the torsional forces by a force scale factor, but the optimization of the force scale factor was computationally demanding—not to mention its temperature dependency. To avoid the necessity of the optimization and to create more realistic force field modifications, the temperature was introduced during the Boltzmann inversion in the following form:

1 $$\mathrm{PE}F_{i}\left(\alpha \right)=-RT\times \ln\left(P_{i}\left(\alpha \right)\right)$$

**Figure 3:**
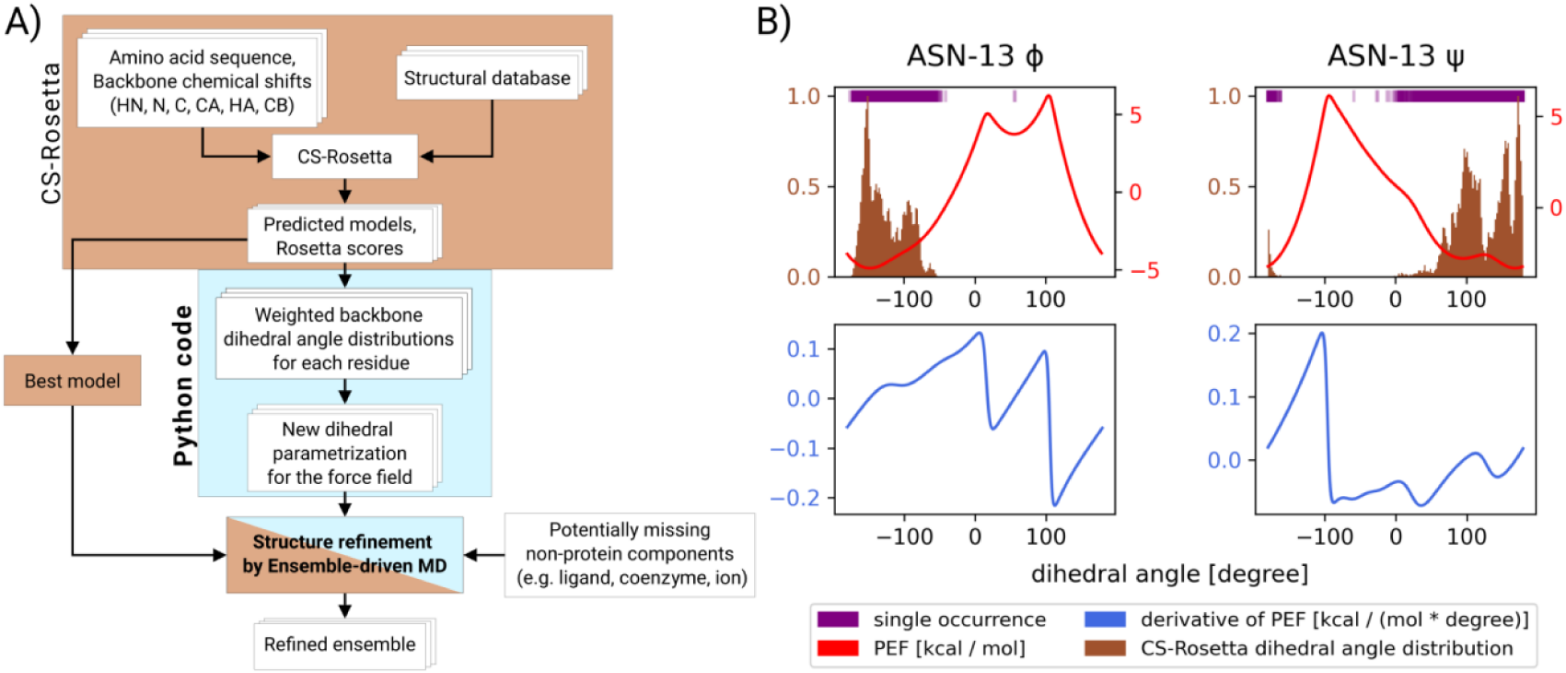
An overview of the Ensemble-driven MD structure refinement method after applying CS-Rosetta. A) The block diagram shows the whole structure determination process. The first phase (brown) is the application of CS-Rosetta to calculate a structure ensemble from the amino acid sequence and the backbone chemical shifts using its structural database. The result of CS-Rosetta is an ensemble of predicted models and related Rosetta scores. Then, our Python3 code (blue) analyzes the results to generate a new dihedral parametrization for the force field. Ensemble-driven MD is then run by using the new force field parameters and the best-scoring CS-Rosetta model as a starting point. The model can also be complemented with the missing nonprotein components before running the EDMD simulation. B) The figure shows the CS-Rosetta Phi and Psi dihedral angle distributions of Asn13 of CRBP as a brown histogram. The single occurrences of the angles are represented as purple lines. The potential energy function (PEF) is depicted as a red line, while its derivative is depicted as a blue line.

Here, *R* is the gas constant, *T* is the temperature, and *P_i_* (α) is the weighted potential density function for the given torsion angle. The first negative derivative of the PEFs (−dPEF) was then taken and used to parametrize each torsional rotation. The schematic representation of the process is shown in a block diagram in Figure 3A, while examples of the torsion angle distribution, PEFs, and dPEFs are depicted in Figure 3B. The best CS-Rosetta model of each system was used as the starting structure for the simulations. The missing small-molecular component(s) were inserted based on the reference structures. In the absence of knowledge of the binding pocket, one must perform docking to insert the nonprotein components. Systems were then solvated in water, and ions were added to set ionic strength and neutralize the overall charge (following the regular MD protocols). Two parallel, 1000 ns-long EDMD refinement simulations were run using the new torsional force field parameters. The second half (500–1000 ns) of each parallel simulation was analyzed as final results. According to the backbone RMSD, all simulations had converged by 500 ns of simulation time or earlier (Figure 4).

**Figure 4:**
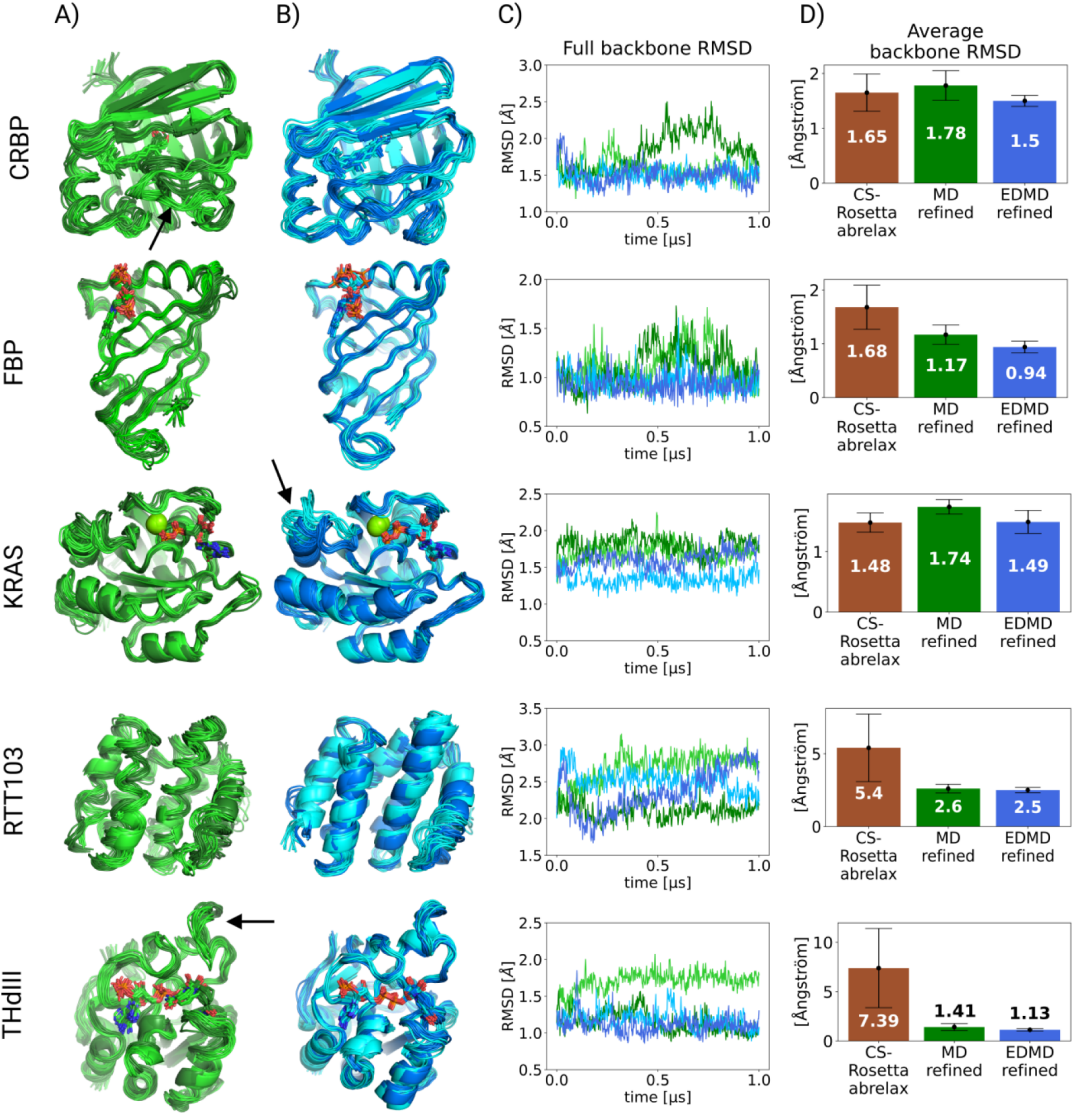
Results of MD refinement (using the original force field with unmodified backbone PEFs) and EDMD refinement results after CS-Rosetta structure determination: A) The 10 models that represent the results of each of the two parallel simulations of the MD refinement (shown in light and dark green, separately). The second halves of the 1000 ns simulations are considered as results. Discrepancies between parallel simulations are labeled by black arrows. B) The 10 models that represent the results of each of the two parallel simulations of the EDMD refinement (shown in light and dark blue, separately). The second halves of the 1000 ns simulations are considered as results. C) Backbone RMSD of each simulation is depicted. D) The average backbone RMSD and its standard deviation are shown for the 10 best CS-Rosetta models (brown), the MD refinement (second halves of the 2 parallels, green), and the EDMD refinement (second halves of the 2 parallels, blue) relative to the X-ray crystal structure. In the case of RTT103, the mean NMR structure was used as a reference in the absence of a crystal structure. The exact percentages for each simulation are shown in Table S1. The largest standard error of the mean of the CS-Rosetta ensembles is about 0.04 Å (sample size is 10000), and approximately 0.001 Å for the MD/EDMD refined data (sample size is 125000), so all the differences in the mean values are significant.

One of the advantages of the EDMD structure refinement method is that it retains the structural information on the CS-Rosetta ensemble that is most directly linked to the experimental chemical shift values, namely the range of possible and probable backbone dihedral angles for each residue. The new torsional force field parameters incorporate these chemical shift-based structural restraints, while a more complex force field is applied instead of the Rosetta scoring function. The MD algorithm also provides a more efficient sampling method to explore the experiment-based conformational space. This is exemplified by the EDMD results in the cases of RTT103 and THdIII, where the CS-Rosetta structure determination was not successful (as the calculations did not converge). Figure 4B shows that EDMD refinement was able to elucidate the correct conformation of these systems. After EDMD refinement, the considered 5 proteins reached conformations similar to their reference structures (see Figure 5A, B). The average backbone RMSD relative to the reference crystal structure decreased during the refinement step in every case, except for KRAS, where it remained similar (Figure 4D or Table S1). The most significant improvement was achieved for RTT103 and THdIII, where the average and standard deviation of RMSD were both greatly reduced (Figure 4C, D). The backbone RMSD improvements compared to the backbone RMSD of the unrefined CS-Rosetta models also translate to heavy-atom RMSD improvements (see Figure S6).

**Figure 5:**
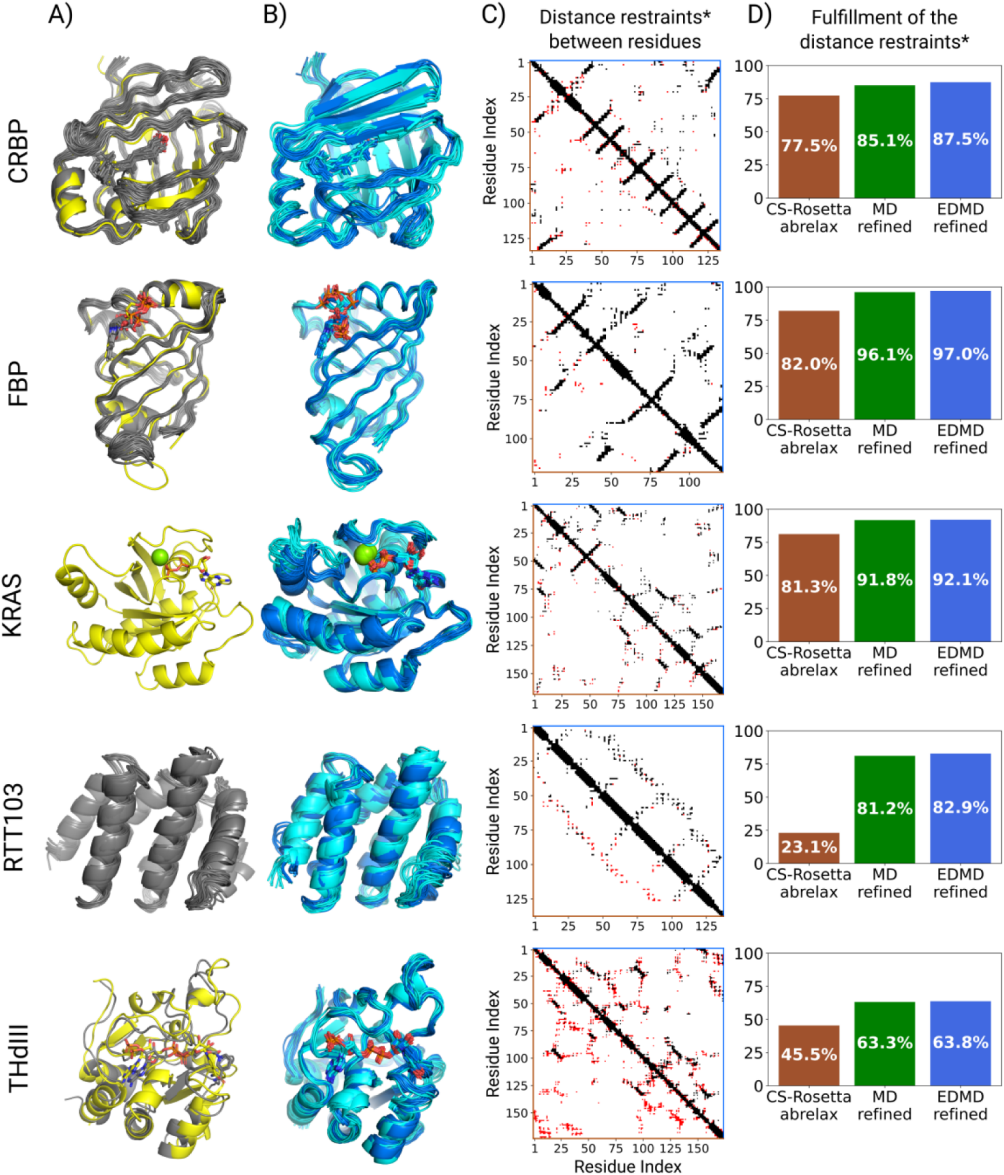
EDMD refined results and the NOE-based validation. A) Reference structure determined by crystallography (yellow) and the NMR reference structures (gray). B) The 10 models that represent the results of each of the two parallel simulations of the EDMD refinement (shown in light and dark blue, separately). The results of the parallel simulations are quite similar, showing that the method is reproducible. The models also contain the nonprotein compartments, which are shown explicitly. C) Asymmetrical binary matrices show whether there is any NOE distance restraint between each residue pair. If a residue pair is closer than 6 Å in the best 10 CS-Rosetta models (brown, lower triangle) or in the EDMD refined ensembles (blue, upper triangle), then the pixel between them is black. If they are not close enough, but there is a measured NOE between them, the pixel is red. If neither, the pixel is white. More information about the definition of these pictures is shown in Figure S5. D) The fulfillment of the NOE distance restraints is depicted in bar diagrams. The CS-Rosetta Abrelax results are shown in brown, the MD refined (using the original force field) is shown in green, and the EDMD refined (dihedral force field parameters derived from experiment) is shown in blue. The exact numbers for each simulation are shown in Table S3. * During the calculation of the percentages, it was only considered whether two residues are close enough to form NOE signals, but not which protons specifically (as in the NOE patterns in Figure 5C).

While the overall structures derived by EDMD were quite similar to the reference structures, we also found two notable differences. In the case of FBP, there is a discrepancy in the reference structures (Figure 1A, black arrow). Gel filtration results^31,41^ indicated that even in the solution phase, the protein is at least partially dimeric, and the crystal structure of FBP contains tightly bound dimers (based on which the PISA server^42^ also estimates solution-state dimerization). However, the referenced NMR structure was solved in the monomeric form. The structures of the monomeric unit derived using the two experimental methods (crystallography vs NMR) are highly similar, with one notable difference: the L3 loop connecting the β4 and β5 strands is in a different conformation. As this is a solvent-immersed loop, NOE restraints were not available to clearly decide if one conformation is more likely than the other when building the NMR structure.^32^ However, in the context of dimerization, this is a functionally significant difference because the loop, in its NMR-derived conformation, overlaps with the FMN binding site of the other monomer—excluding dimer formation. The CS-Rosetta algorithm relies on a structural database containing a wide variety of proteins, where segments with similar sequences and chemical shifts may appear in different environments; therefore, the reference structure set will sample the likely behavior of the given loop in different settings, slightly biased toward its stabilized conformations (since freely fluctuating loops do not produce interpretable electron density and thus will not appear in crystal structures). If no such stabilized conformation of a segment was previously found, then the CS-Rosetta ensemble will be ambiguous in this region, providing wide and shallow PEF minima for the implicated backbone torsions, allowing EDMD to freely sample all accessible conformations and resulting in a conformationally diverse ensemble. The EDMD models derived by us for the implicated loop of FBP are well-defined and quite similar to the conformation seen in the crystal structure. This means that there are conformations that are compatible with the measured chemical shifts that also happen to place this loop in a conformation that allows dimer formation—even though this aspect was not included in the EDMD simulations. Thus, our results indicate that the chemical shifts measured in this case could also support the dimeric form or a monomer that is readily compatible with dimerization.

Interesting differences are also present in the EDMD-derived structures of THdIII too. According to Sundaresan et al.,^38^ loop D has two conformations in the NADP-bound state. In the open conformation (PDB ID: 1PNO chain A), the NADP is more exposed to water, while in the closed conformation (PDB ID: 1PNO chain B), the ligand interacts with some highly conserved side chains of the loop D region. In the reference crystal structure (PDB ID: 1PNO, chain B) and the NMR structure (PDB ID: 1E3T), loop D regions are both clearly, although not identically, in the open conformation. It is also important to note that, at the atomic level, the two reference structures show some differences in general (see Figure 1A, black arrow). The EDMD-refined models are in the open conformation, which is expected as the CS-Rosetta ensemble was generated using the chemical shifts of the reference NMR structure. However, the EDMD-refined models are more similar to the crystal structure than the NMR structure (average backbone RMSD of the 2 parallel EDMD refinements, 500–1000 ns, is 1.1 ± 0.1 Å and 2.6 ± 0.1 Å, relative to the reference crystal structure and the reference NMR structure) in the case of this system as well, just as seen previously.

Another important feature of EDMD refinement is that it provides the means to reinsert nonprotein components. Prosthetic groups, cofactors, ligands, and substrates are all essential protein components that play a decisive role in their structure and functionality. The absence of these moieties during structure calculation can significantly distort the binding pocket and its immediate environment, as well as further regions through allostery. In all four systems where such a partner was present during structure determination, the reinsertion of the nonprotein component(s) by EDMD was successful. The proteins formed the expected interactions with the small molecules (and with Mg^2+^ in KRAS). The calculated positions and conformations of the cofactors also resemble those of the reference structures.

But in the cases of FBP and THdIII, there are, again, some subtle but significant differences between the NMR and crystal structures with respect to the binding mode of the partner molecules. The binding pockets of each system are shown in Figures S1–4. The conformation at the beginning of the ribityl side chain of FMN in the reference NMR models shows a slightly different binding mode relative to the reference crystal structure (see Figure S2). According to Otting et al.,^32^ the FMN ligand is bound to a shallow groove, where the isoalloxazine ring is well-described in their NMR models. Meanwhile, no NOE signals could be identified in connection with the terminal part of the ribityl side chain, so it is thought to be disordered. The authors even proposed that the negatively charged phosphoester group at the end of the ribityl chain might interact with the positively charged N-terminus of the α2-helix (as it is in the crystal structure). The EDMD-refined models are more similar to the reference crystal structure in terms of the interactions between the ribityl side chain and the protein. However, the very end of the ribityl side chain is lot more disordered than in any of the references, which seems logical in the absence of NOE signals.

The nicotinamide ribose conformation in THdIII also differs in the NMR reference model, relative to the crystallographically determined reference structure. The models refined by EDMD are very similar to the crystal structure in this system as well (Figure S4).

In Figure 4, it is also apparent that the EDMD method is robust: parallel simulations are not strictly necessary (although they might uncover hidden degrees of freedom or minor, sparsely sampled conformations). The most noticeable difference between parallel refinements occurs in the angle of inclination of the switch-II region of KRAS (residues 57–76), which is known for being very flexible^34^ (see Figure 4B black arrow).

### 2.3 Validation by NOE Distance Restraints

Distance restraints from NOESY-type experiments are available for all of the systems under investigation. However, they were not used in deriving the CS-Rosetta ensembles or our EDMD-based models; therefore, they can be used for validation and for comparing the different approaches utilized in this study. To have an easily interpretable overview of the fulfillment of the NOE-based distance restraints, asymmetric binary matrices were created (see Figure 5C) which are colored according to whether each residue pair meets the measured distance restraints or not. Black pixels between residue pairs indicate that some protons of the residues are close enough on average to form NOE signals (6 Å), while the red pixels indicate residue pairs where this is not the case. A detailed explanation of the creation of the matrices is shown in a block diagram in Figure S5. This comparison clearly demonstrates that the EDMD-refined models are more consistent with distance restraints than the unrefined, CS-Rosetta-generated ensembles —even though the restraints have not been used in the structure calculation process.

More than 10% improvement in NOE fulfillments was achieved by the EDMD refinement (blue bars) in all of the studied cases, as compared to applying the CS-Rosetta Abrelax protocol alone (which could not reach 85%). Figure 5D also shows that MD refinement using an unmodified force field and the CS-Rosetta—provided starting structure also produces a significant improvement in the number of fulfilled NOE restraints, but slightly less so than EDMD simulations. The differences between the MD and EDMD simulations, and the advantages of our EDMD method, will be discussed later in detail.

RTT103 and THdIII were the two systems for which the CS-Rosetta protocol did not converge. This can be seen in Figure 2B as well, where both of these structural ensembles show great heterogeneity. The corresponding NOE-based quality checks (Figure 5C, D) also reflect suboptimal results: a great many outliers can be seen, and low fulfillment ratios of 21.3% and 45.5% in the case of RTT103 and THdIII, respectively. On the other hand, the distance profile of the EDMD-refined results (Figure 5C, blue, upper triangle) covers the measured NOE patterns. EDMD simulations resulted in considerable improvement in both cases and a structural ensemble that is a much better description of these challenging systems as well. The relatively low success rate in the case of THdIII is—at least partly—caused by the unavailability of the original NOE data. The “measured NOE distance restraint” set was approximated from a single NOE-based structure (PDB ID: 1E3T). Since the flexibility of the system could not be described in a single model, a lot more NOEs were predicted than are usually present in similarly sized molecules, since they could not average out.

### 2.4 Structure Refinement and NOE Validation for NOE-RASREC-Rosetta

We were also curious about the efficiency of our newly developed method, EDMD, in the case of structure calculations where distance restraints were also applied. The structures of the two systems, those of CRBP and FBP, were recalculated using the CS-Rosetta RASREC protocol supplemented with NOE distance restraints (we will refer to these as NOE-RASREC-Rosetta). 100 models were generated using this protocol, and, similarly as in the case of the Abrelax protocol, the 10 best-scoring models were used as final results. The NOE-RASREC-Rosetta ensembles and their EDMD refinement are summarized in Figure S7. The NOE-RASREC-Rosetta structure elucidations were successful. The best models (Figure S7B) are quite like the references, except for the absence of nonprotein moieties.

EDMD refinements were carried out for these model systems as well, just as in previous cases. The EDMD-refined models are shown in Figure S7C, which are similar to the NOE-RASREC-Rosetta models, but they also contain cofactor molecules (RTL and FMN for CRBP and FBP, respectively). The interaction network between the small molecules and the protein, and the conformations of the RTL and FMN, are similar to those seen in the reference crystal and NMR structures (the binding pockets are shown in Figures S1 and S2).

The NOE distance restraint patterns (Figure S7D) and fulfillment ratios (Figure S7E) were also calculated. Although the difference between the NOE-RASREC-Rosetta results and the EDMD results is more modest, the quality of the structures still gets better in the course of EDMD refinement. The NOE fulfillment ratio improves from 80.6% to 89.5% in the case of CRBP, and from 86.2% to 96.4% in the case of FBP.

The convergence of the refinement is shown as backbone RMSD plots in Figure S8. The average backbone RMSD (relative to the reference X-ray structure) has not significantly changed after the refinement, which is because, due to the applied NOE-based distance restraints, the NOE-RASREC-Rosetta calculations have already found the appropriate conformation of the proteins. It can also be observed that the standard deviation of the backbone RMSD has significantly decreased after the refinement.

### 2.5 The EDMD Force Field is Conformation Specific

To compare the performance of the introduced EDMD methodology using new experiment-based dihedral force field parameters and the original force field, regular MD simulations were also performed with unchanged force field parameters. Here, we present three examples to highlight the advantages of EDMD: refinement of CRBP and THdIII from the CS-Rosetta models, and refinement of FBP from the NOE-RASREC-Rosetta models.

#### 2.5.1 CRBP

In the case of CRBP, the 2 parallel EDMD simulations resulted in similar results (with 1.5 ± 0.1 and 1.5 ± 0.1 Å average backbone RMSD relative to the reference crystal structure); however, the same was not the case for the MD simulations (with 1.6 ± 0.1 and 2.0 ± 0.2 Å). In the latter, one of the MD parallels (MD-2), a distinct EF-loop conformation appears, penetrating the hydrophobic pocket much deeper than expected (see the whole models in Figure 3A and the binding pocket in Figure S1). The flexibility of this region has long been noted,^43^ since the EF-loop, together with the CD-loop and a short helix, forms the portal region of CRBP that allows ligand binding and release. However, the conformations sampled by MD-2 do not occur in any of the reference structures and seem to be very unlikely based on the results of Li et al.^30^ Li et al. assigned NOEs between the ionone ring protons (H16*/H17*) of RTL and the Ile-77 side chain (within the EF-loop), which is not close enough to this part of the polyene chain in the trajectory in question (MD-2). Interestingly, in the EDMD-generated ensemble and also in that of MD-1, RTL lies in the expected orientation within the pocket, with the ionone ring close to Ile-77, but also not quite close enough for the protons in question to form NOE signals. Nevertheless, the less optimal results of MD-2 are also reflected in an overall decrease in the fulfillment of NOE restraints: only 84.8% of the residue pairs that are expected to be close enough on average to form NOE signals are indeed within interacting range, while 86.5% for MD-1, 88.1% for EDMD-1, and 87.9% for EDMD-2 (Table S3). The Lys-40 and Gln-108 side chains, which play an important role in inducing binding affinity and specificity,^29^ have a similar conformation like the reference structures, interacting with the hydroxyl group of RTL in all the refined models. The case of CRBP exemplifies that by modifying the torsional force field parameters in the EDMD refinement, the force field becomes conformation specific, supporting those conformations that were present in the experimentally determined structural ensemble.

#### 2.5.2 THdIII

During the refinement of THdIII, one of the MD simulations reached a slightly different result (Figure S4). The first parallel of the MD simulations (MD-1) stands out by having greater RMSD relative to both the crystallographically determined and NMR reference structures (see Tables S1 and S2) and a lower ratio of distance restraints fulfillment (see Table S3) than MD-2 and both EDMD refinements. Based on the NOE measurements of Jackson et al.,^39^ Tyr-26 (HD*/HE*) should interact with the adenosyl H8 proton of the NADP, but the average distance between the heavy atoms is 8.4 ± 0.8 Å in the MD-1 trajectory, while it is 4.7 ± 0.5 Å in that of MD-2, and 4.7 ± 0.6 Å and 4.6 ± 0.6 Å in the EDMD trajectories. Heavy atom distances were also measured in the reference structures, as shown in Table S4. According to Stout et al.,^38^ the Asp-103 and the nicotinamide ribose should be close to each other, but the side chain carboxyl group in the MD-1 refinement is too far to form an interaction (heavy atom distance is 8.3 ± 0.4 Å, while it is 2.6 ± 0.1 Å in both MD-2, EDMD-1, and EDMD-2) (Table S4). The amide group of the nicotinamide ring of NADP is cradled by loop B in both reference structures, but in the MD-1 refinement, the nicotinamide ring is in a different conformation. It was also described^38^ that the Tyr-142-Trp mutation accelerates the NADP/NADPH release, so this residue must also be important for the coordination of the coenzyme. In the MD-1 refinement, the NADP nicotinamide ring forms an offset stack instead of an edge-to-face interaction with the aromatic ring of Tyr-142. The results concerning THdIII show that by using an experiment-based force field specific for the given protein backbone, undesired conformational changes can be avoided, not just for the protein, but for the nonprotein moieties as well, as they interact closely with each other.

#### 2.5.3 FBP

In the third example, during the MD-1 refinement of the FBP-FMN complex (from NOE-RASREC-Rosetta), the α2-helix and the preceding loop (residues 49–51) undergo some minor conformational deviation, along with the ribityl side chain of the FMN molecule (see Figure S2). Suto et al.^31^ described a similar FMN-binding pocket (to the EDMD-1, EDMD-2, and the MD-2 trajectories) based on their crystallographically determined structure, emphasizing that next to the FMN ligand, the side chain of Met-51 runs parallel with the ribityl group toward the 2,4-pyrimidinedione ring. The FMN binding pattern derived by the EDMD-1, EDMD-2, and the MD-2 simulations is highly similar to these, but the results of MD-1 suggest a different FMN side chain conformation that is not compatible with the above-described mode. In this trajectory, the ribityl side chain occupies the location of the Met-51 side chain, which flips toward the opposite direction, to the solution. Because of the resulting conformational rearrangement, the loop between β3 and α2 is too far from the ribityl side chain to form NOE signals. Heavy atom distances are 11.4 ± 1.1 Å in MD-1, while they are 3.1 ± 0.4 Å in MD-2, 3.8 ± 1.2 Å in EDMD-1, and 4.4 ± 1.5 Å in EDMD-2 (see the values for the reference structures and more measurements in Table S5). The higher average backbone RMSD values relative to both reference structures (Tables S1 and S2) and the lower ratio of NOE distance restraint fulfillments (Table S3) all support the inaccuracy of the conformational change presented by the MD-1 refinement, which is avoided by both EDMD trajectories.

The FBP-refined models were also studied by LoCoHD^44^ (local composition Hellinger distance), which is capable of comparing environments based on chemical composition. During the conformational change of a residue or a ligand, the chemical properties of its surroundings also change, which can be effectively probed by using this metric. The rearrangement of the Met-51 side chain is nicely represented by the elevated value (and standard deviation) of the LoCoHD of the Sδ (delta sulfur) atom (Figure S9C). We have also calculated higher LoCoHD values for the atoms of FMN (namely, the P phosphorus and the C2 aromatic carbon atoms) (Figure S9A, B). While the LoCoHD of P of FMN is about 0.1 and 0.25 in MD-2 and both EDMD simulations, it reaches 0.35 in the MD-1 trajectory, highlighting the difference in the chemical compositions.

This reaffirms that the EDMD method provides an opportunity to drive the refinement simulation toward favorable conformations, which are characteristic of the experimentally determined structural ensemble. These examples clearly emphasize the advantages of EDMD compared with traditional MD simulations. The case of THdIII and FBP even shows that, through the new experiment-based torsional force field parametrization, the improper conformational changes of the small molecules can also be prevented because of the close interaction of the protein and the nonprotein moiety.

## 3 Discussion

In a previous work,^28^ we have introduced a technique that makes it possible to analyze the dihedral (or torsional) angle distribution of a structural ensemble to define continuous, differentiable potential energy functions (PEFs). The new PEFs can replace the torsion angle potentials of the original force field (AMBER-ff99SBildnp*^45^) to perform chemical-shift-driven molecular dynamics (csdMD) simulation as structure refinement, without predicting chemical shifts after every step of the MD simulation. In this original approach, during the definition of the PEFs, the standard deviations of the PEFs were scaled to match the standard deviation of the corresponding torsion angle potential term in the original force field. The negative first derivative of the PEF is the force acting on the torsion angle during a csdMD simulation.

We present an improved version of the Ensemble-driven MD (EDMD) method and demonstrate its effectiveness on a benchmark set of 5 proteins ranging from 13.1 to 19.2 kDa, which cover all globular CATH^40^ classes (mainly alpha, mainly beta, and alpha-beta), four of which contain nonprotein moieties. As an improvement, instead of scaling the PEFs to the original force field, the temperature factor (in our experiments, set to *T* = 310 K) was introduced during the definition of PEFs, creating a more realistic new force field parametrization. The structural ensembles of the benchmark set were generated by CS-Rosetta using only chemical shifts; then the missing nonprotein moieties were reinserted into the best model based on the reference structure, and EDMD refinement simulations were run. If no reference structure exists, the binding pocket can be found by docking, and refinement can be run afterward. The structure refinement was successful in every case, even if the CS-Rosetta structure determination had not converged: the average backbone RMSD improvement was 0.3 Å for the converged and 4.6 Å for the nonconverged models. The nonprotein components were also successfully reinserted, forming realistic interactions with the protein, like in the references. NOE-derived distance restraints were available for all the systems but were only used for validation. On average, the fulfillment of protein–protein distance restraints improved by 12% for converged models and 39% for nonconverged models, thanks to EDMD refinement.

The performance of the EDMD method was studied for the iterative protocol of RASREC-Rosetta, supplemented with NOE-derived distance restraints, in the case of 2 small molecule-containing proteins. Although the backbone RMSD improvements were less significant due to the higher quality of the original models, almost 10% improvement was achieved in distance restraint fulfillment, despite the fact that the EDMD simulation was only restrained by the new backbone dihedral angle parametrization.

Unrestrained MD simulations were also run, and minor conformational alterations occurred in both the protein and the small molecules, which were proven to be wrong. The CS-Rosetta ensemble-derived new force field parametrization is specific for the backbone conformations present in well-scoring models, preventing such conformational changes in EDMD refinements.

Although the EDMD structure refinement strategy was tested for CS-Rosetta calculations, the method can be used to refine any structural ensemble. The backbone torsional angles of any ensemble can be analyzed in a similar manner to generate continuous, differentiable potential energy functions from discrete data. Providing scores or energies for the structures allows one to tune the contribution of single models to the definition of potential energy functions. Creating new torsional parametrization and running an EDMD simulation allows the protein conformation to effectively explore the local minima of the φ/ψ phase space, utilizing a more complex force field while enabling the inclusion of nonprotein components.

## 4 Materials and Methods

### 4.1 Proteins Under Investigation

In this study, the CS-Rosetta calculations were carried out using the same chemical shifts as those for the NMR reference structures. The Biological Magnetic Resonance Data Bank (BMRB) entries are BMR5578 for CRBP, BMR30327 for RTT103, and BMR4236 for THdIII. The chemical shift data for FBP was accessed from the PDB directly. For the KRAS NMR data, chemical shift data from our own measurements was applied (BMR53329). The reference X-ray crystal structures and NMR structures from the PDB are 5LJB and 1MX8 for CRBP, chain A of 1FLM and 1AXJ for FBP, chain A of 4OBE for KRAS, 5WOZ for RTT103, and chain B of 1PNO and 1E3T for THdIII. As for their size, CRBP has 134 residues and is 15.7 kDa, FBP consists of 122 amino acids and is 13.1 kDa, KRAS has 169 residues and is 19.2 kDa, RTT103 has 138 residues and is 16.1 kDa, and THdIII has 174 amino acids and is 18.7 kDa protein mass.

### 4.2 NMR Measurements and Chemical Shift Assignment for KRAS

NMR samples contained about 0.8 mM KRAS, 10 mM MgCl_2_, 3 mM NaN_3_ in PBS, 7% D_2_O, and 0.01 mM DSS at pH = 7.4. [^15^N,^1^H]-BEST-TROSY, [^13^C,^1^H]-HSQC, and 3D NOESY-[^15^N,^1^H]-sfHMQC were recorded on a Bruker Avance Neo 950 NMR spectrometer equipped with a 5 mm TCI cryo ^1^H,^15^N,^13^C Z-GRD probe. 3D NOESY-[^13^C,^1^H]-HSQC was recorded on a Bruker Avance Neo 1200 NMR spectrometer equipped with a 3 mm TCI cryo ^1^H,^15^N,^13^C Z-GRD probe. 3D [^15^N,^1^H]-BEST-TROSY-HN­(CO)­CACB, [^13^C,^1^H]-SOFAST-HMQC aromatic, and 3D NOESY-[^13^C,^1^H]-SOFAST-HMQC aromatic were recorded on a Bruker Avance Neo 900 NMR spectrometer equipped with a 5 mm TCI cryo ^1^H,^15^N,^13^C Z-GRD probe. 2D NOESY, 3D [^15^N,^1^H]-BEST-TROSY-HNCO, and 3D [^15^N,^1^H]-BEST-TROSY-HN­(CA)­CO were recorded on a Bruker Avance III HD 800 NMR spectrometer equipped with a 5 mm TCI cryo ^1^H,^15^N,^13^C Z-GRD probe. 3D H­(CCC-TOCSY)­(CO)­NH, 3D (H)­C­(CC-TOCSY)­(CO)­NH, and 3D [^15^N,^1^H]-BEST-TROSY-HNCACB were recorded on a Bruker Avance III HD 700 NMR spectrometer equipped with a 5 mm QCI cryo ^1^H,^15^N,^13^C,^31^P Z-GRD probe. Measurements were performed at 298 K. The temperature was calibrated against a standard methanol solution. ^1^H chemical shifts were referenced with respect to the ^1^H resonance of the internal DSS, whereas ^13^C and ^15^N chemical shifts were referenced indirectly by using the corresponding gyromagnetic ratios according to the IUPAC convention. All spectra were processed with Bruker TOPSPIN 3.6 and TOPSPIN 4.1.

We have performed a semiautomatic full chemical shift assignment for KRAS, which consisted of four steps. In the first step, ARTINA^11^ structure calculation was carried out on the NMRtist platform, utilizing all the measured spectra and the amino acid sequence. In the second step, the peak picking and assignment for the backbone chemical shifts were manually corrected in CcpNmr 3.2.0. As a third step, the ARTINA structure calculation protocol was run again, supplemented with the shift list of the ^1^H–^15^N HSQC. As a final step, the results were checked in CcpNmr again.

### 4.3 CS-Rosetta Structure Determination

Chemical-Shift-Rosetta version 3.6 and Rosetta release version 2021.16 were applied. For the CS-Rosetta calculations using the Abrelax protocol, only the protein sequence and the chemical shifts were used. For the NOE-RASREC-Rosetta structure elucidation, the RASREC^18^ protocol was applied, and NOE-derived distance restraints were also used. During the fragment library building process, the inclusion of homologous proteins was allowed (the “–nohom” flag was not applied for fragment picking). 10,000 models were generated from CS-Rosetta and 100 from NOE-RASREC-Rosetta calculations.

### 4.4 EDMD Refinement

To perform EDMD refinement, the Python3 codes of the EDMD package (https://github.com/gadaneczm/EDMD_package) were run to analyze the backbone torsional angle distributions for the structural ensembles from CS-Rosetta and NOE-RASREC-Rosetta. The Rosetta scores were also used to generate weighted angle distributions and PEFs in a similar way to that used before. The only important difference between CS-Rosetta and NOE-RASREC-Rosetta calculations was the number of models in the ensemble, but this issue is addressed by our scripts by adjusting the width of the Kernel function based on the number of structures inside the ensemble. The Python3 codes created tabulated potentials for each of the φ and ψ torsion angles (in the form of separate XVG files) and a related topology file (NEW.TOP). The initial models for refinement were built from the best CS-Rosetta (or NOE-RASREC-Rosetta) models. The missing nonprotein moieties were added based on the reference crystal structures; the crystal structure was aligned to the best ensemble model, and the position of the ligand was transferred in this way to the polypeptide structure. The models were solvated in water using the OPC water model,^46^ and the systems were neutralized with sodium and chloride ions at physiological salt concentration (0.15 M). GROMACS version 2022.2 ^47^ and the AMBER-ff99SBildnp*^45^ force field were used for the EDMD simulations. We applied ACPYPE^48,49^ (AnteChamber PYthon Parser interfacE v. 2023.10.27) to generate the force field-specific parametrization of the RTL, FMN, and NADH small molecules. The parametrization from Steinbrecher et al.^50^ was used for GDP. The equilibration of the systems consisted of the following steps: first, a steepest descent integrator with position restraints of 1000–500, 500–100, 100–0, and then 0–0 kJ × mol^–1^ × nm^–2^ (for the protein and the nonprotein moieties, respectively) and a maximal force tolerance of 50 kJ × mol^–1^ × nm^–1^ was used. Next, an NVT equilibration was conducted using the leapfrog integrator for 50,000 steps with a 2 fs step size with position restraints of 1000, 500, 100, and 0 kJ × mol^–1^ × nm^–2^ at 310 K. Finally, an unconstrained NPT step was followed to allow the introduction of pressure. The EDMD simulations were performed at 310 K for a total of 1 μs with two parallel simulations. To explore the temperature dependence of the protocol, EDMD simulations of the KRAS system were repeated at 350 K also (see Figure S10).

### 4.5 MD Refinement

The MD refinement simulations were carried out in a similar way to the EDMD, but without force field modifications.

### 4.6 Trajectory Analysis and Plotting

The figures of the protein structures were created with PyMol 3.1.4.1. The analysis of the trajectories (like RMSD and distance calculations for NOE predictions) was carried out by MDAnalysis.^51,52^ The results were plotted using Matplotlib. The solvent-accessible surface area (SASA) values of the ensembles were calculated using GROMACS version 2022.2 ^47^ and are summarized in Table S6. Clustering for Figure S10 was performed using GROMACS version 2022.2 ^47^ with the GROMOS method and a cutoff of 1.0 Å.

LoCoHD^44^ v0.2.0 was applied. The all-atom primitive typing scheme was used and supplemented with the primitive types of the FMN ligand: aliphatic and aromatic carbons, neutral nitrogens, and neutral oxygens were classified according to the corresponding primitive types defined for protein atoms, while a new primitive type was defined for the phosphorus. For anchor atoms, FMN C2 and P atoms and Met-51 Sδ were selected. The tag pairing rule was set to accept_same = True, meaning that atoms of the same residue were included in the environment. The default weight function was used. No primitive type weighting was employed.

## Supplementary Material



## Data Availability

The NMR chemical shift assignment for KRAS is available in the BMRB (https://bmrb.io/, accessed in September 2025) under entry 53329. The structure ensembles generated by CS-Rosetta and the necessary files for the reproduction of the structure refinements is collected and available in a Figshare repository with an accession code of 10.6084/m9.figshare.30157474.v1. Python code for the EDMD project is available at the GitHub repository https://github.com/gadaneczm/EDMD_package. A release version of 2.0.0 was used for this study.

## Notes

Figure S1, Overview of CRBP refinements and the RTL-binding pocket; Figure S2, Overview of FBP refinements and the FMN-binding pocket; Figure S3, Overview of KRAS refinements and the GDP-binding pocket; Figure S4, Overview of THdIII refinements and the NADP-binding pocket; Figure S5, Block diagram illustrating the creation of the asymmetric matrices of the NOE fulfillments; Figure S6, Linear relationship between backbone and heavy-atom RMSD improvements relative to the reference crystal structures and mean NMR structures; Figure S7, Summary of the NOE-RASREC-Rosetta structure determinations and their EDMD refinements; Figure S8, Results of MD refinement (using the original force field) and EDMD refinement results after the NOE-RASREC-Rosetta structure determination; Figure S9, Local composition Hellinger distances (LoCoHD) in FBP refinement simulations based on NOE-RASREC-Rosetta calculations; Figure S10, The temperature dependence of EDMD simulations of KRAS; Table S1, Average backbone RMSD of each CS-Rosetta, MD, and EDMD calculations relative to the crystallographically determined reference structures; Table S2, Average backbone RMSD of each CS-Rosetta, MD, and EDMD calculations relative to the NMR reference structures; Table S3, Fulfillments of the NOE distance restraints in the whole protein for each CS-Rosetta, MD, and EDMD calculations; Table S4, Intermolecular heavy atom distances in THdIII in the reference structures and refined models based on the CS-Rosetta calculations; Table S5, Intermolecular heavy atom distances in FBP in the reference structures and in the refined models based on the NOE-RASREC-Rosetta calculations; Table S6, Average solvent-accessible surface area of each CS-Rosetta, MD and EDMD calculations (PDF)

## Abbreviations

Å: Ångström
CRBP: Cellular retinol-binding protein
FBP: Flavin mononucleotide-binding protein
BMRB: Biological Magnetic Resonance Data Bank
CATH: Protein structure classification database
CS-Rosetta: Chemical-Shift-Rosetta
csdMD: Chemical-shift-driven molecular dynamics
EDMD: Ensemble-driven molecular dynamics
FMN: Flavin mononucleotide or riboflavin-5′-phosphate
GDP: Guanosine diphosphate
KDE: Kernel density estimation
KRAS: Kirsten rat syndrome virus homologue protein
MC: Monte Carlo
MD: Molecular dynamics
NADP: Nicotinamide adenine dinucleotide phosphate
NMR: Nuclear magnetic resonance spectroscopy
NOE: Nuclear Overhauser effect
NOESY: Nuclear Overhauser effect spectroscopy
PDB: Protein Data Bank
PEF: Potential energy function
RASREC: Resolution-adapted structural recombination
RDC: Residual dipolar coupling
RMSD: Root mean square deviation
RTL: retinol
RTT103: Regulator of Ty1 transposition protein
SASA: Solvent-accessible surface area
THdIII: NADP­(H) binding component (domain III) of proton-translocating transhydrogenase
TOCSY: Total correlation spectroscopy
