# Supplementary material for: Improving Protein Structure Determination by Integrating Ensemble-Driven Molecular Dynamics with Chemical Shift-Based Restraints
Source: J Chem Inf Model. 2026 Feb 16;66(5):2844–56. doi: 10.1021/acs.jcim.5c02358 (PMC12977052; doi:10.1021/acs.jcim.5c02358)
Supplement: Supplementary file 1 [file ci5c02358_si_001.pdf]

Supporting Information

Improving Protein Structure Determination by Integrating Ensemble-Driven Molecular Dynamics with Chemical Shift-Based Restraints

*Márton Gadanecz[1,2], Zsolt Fazekas[1], Dóra K. Menyhárd[1,3], András Perczel[1,3,] **

[1] Laboratory of Structural Chemistry and Biology, Institute of Chemistry, Eötvös Loránd University, Pázmány Péter stny. 1/A, H-1117 Budapest, Hungary

[2] Hevesy György PhD School of Chemistry, Eötvös Loránd University, Pázmány Péter stny. 1/A, H-1117 Budapest, Hungary

[3] HUN-REN–ELTE Protein Modelling Research Group, Eötvös Loránd Research Network (ELKH), Pázmány Péter stny. 1/A, H-1117 Budapest, Hungary

*perczel.andras@ttk.elte.hu

Contents

Figure S1 – Overview of CRBP refinements and the RTL-binding pocket

Figure S2 – Overview of FBP refinements and the FMN-binding pocket

Figure S3 – Overview of KRAS refinements and the GDP-binding pocket

Figure S4 – Overview of THdIII refinements and the NADP-binding pocket

Figure S5 – Block diagram illustrating the creation of the asymmetric matrices of the NOE fulfillments

Figure S6 – Linear relationship between backbone and heavy atom RMSD improvements relative to the reference crystal structures and mean NMR structures

Figure S7 – Summary of the NOE-RASREC-Rosetta structure determinations and their EDMD refinements

Figure S8 – Results of MD refinement (using the original force field) and EDMD refinement results after the NOE-RASREC-Rosetta structure determination

Figure S9 – Local composition Hellinger distances (LoCoHD) in FBP refinement simulations based on NOE-RASREC-Rosetta calculations

Figure S10 – The temperature dependence of EDMD simulations of KRAS

Table S1 – Average backbone RMSD of each CS-Rosetta, MD and EDMD calculations relative to the crystallographically determined reference structures

Table S2 – Average backbone RMSD of each CS-Rosetta, MD and EDMD calculations relative to the NMR reference structures

Table S3 – Fulfillments of the NOE distance restraints in the whole protein for each CS-Rosetta, MD and EDMD calculations

Table S4 – Intermolecular heavy atom distances in THdIII in the reference structures and refined models

Table S5 – Intermolecular heavy atom distances in FBP in the reference structures and in the refined models based on the NOE-RASREC-Rosetta calculations

Table S6 – Average solvent-accessible surface area of each CS-Rosetta, MD and EDMD calculations

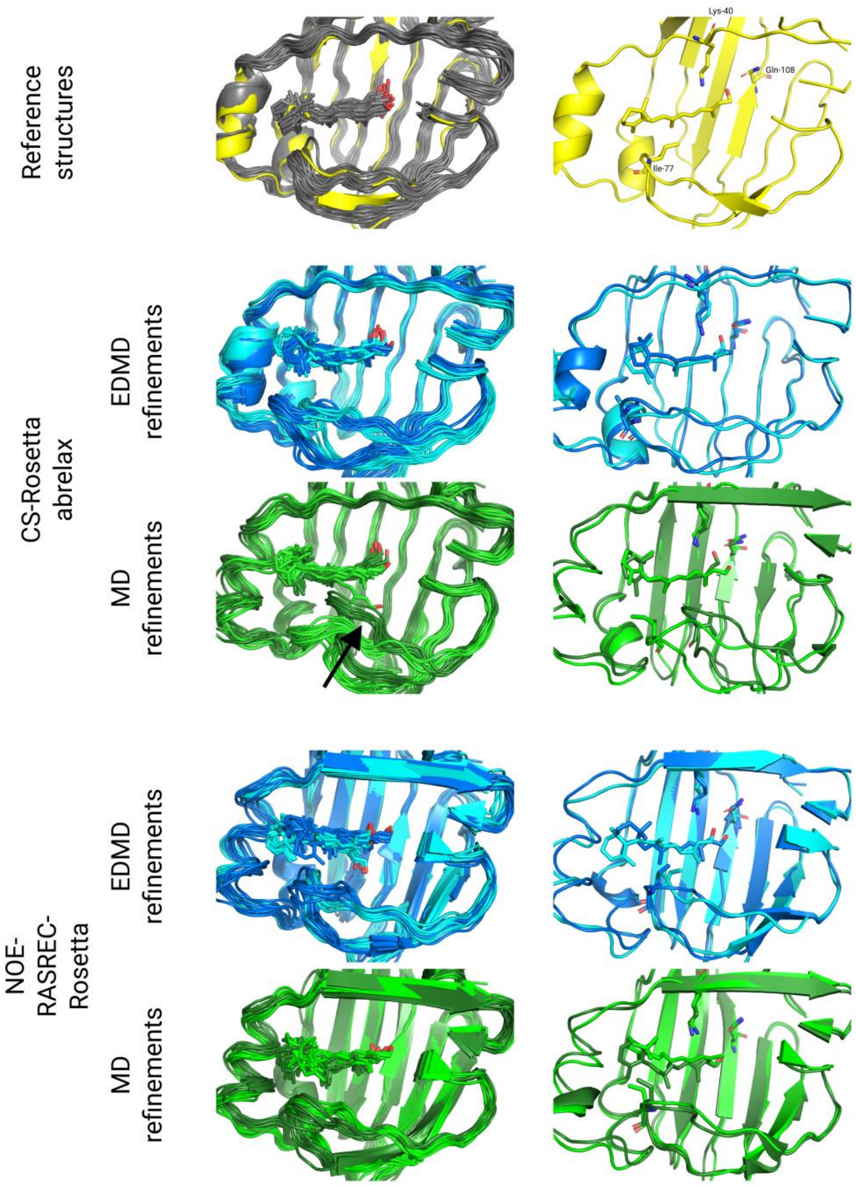

Figure S1. Overview of CRBP refinements and the RTL-binding pocket. The reference crystal structure is yellow (PDB ID: 5LJB), reference NMR models are gray (PDB ID: 1MX8), EDMD refined models are blue (light and dark blue as the first and second parallel, respectively) and unrestrained MD refined models are green (light and dark blue as the first and second parallel, respectively). Residues 50-62 are hidden, so the binding pocket can be seen. The left column shows 10 representative models from the second halves of the trajectories, while the centers of the most populated clusters are shown on the right column. The most important sidechains of the binding pocket are also shown in the cluster centers. The black arrows indicate minor conformational changes in the refinements, which deviate from the reference structures.

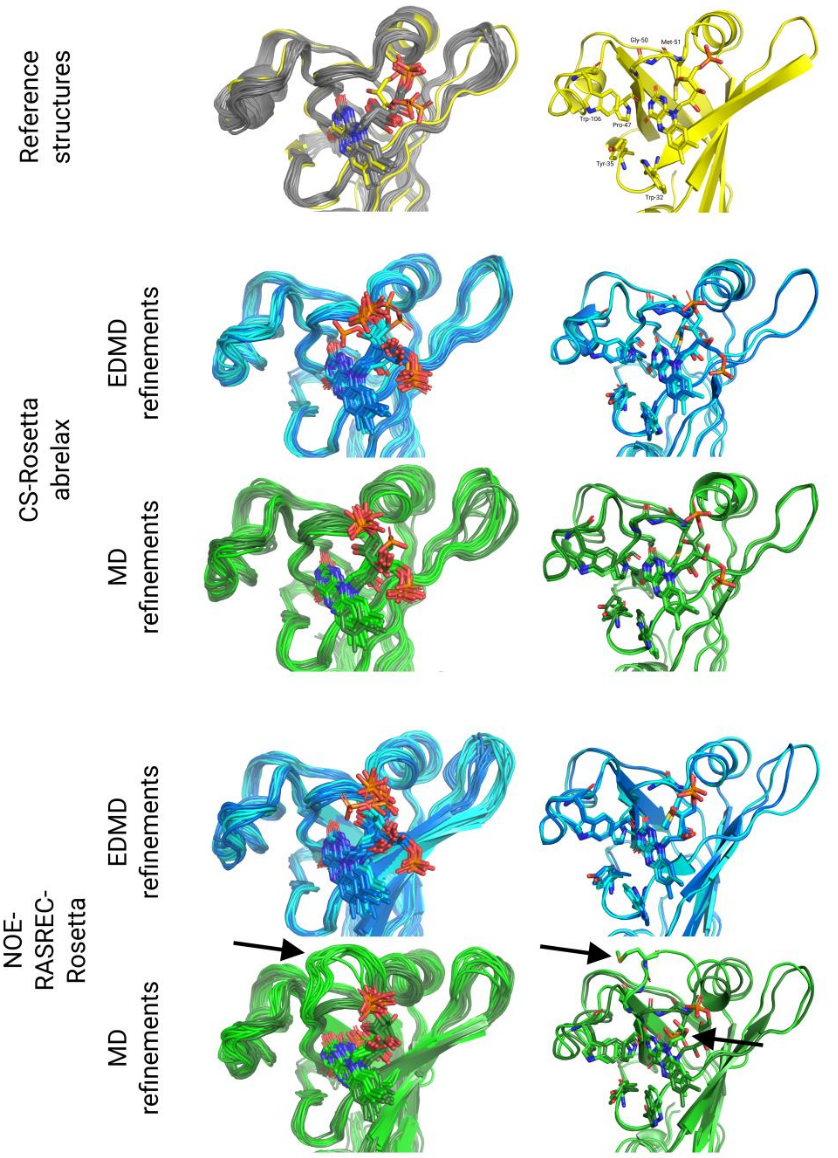

Figure S2. Overview of FBP refinements and the FMN-binding pocket. The reference crystal structure is yellow (PDB ID: 1FLM, chain A), reference NMR models are gray (PDB ID: 1AXJ), EDMD refined models are blue (light and dark blue as the first and second parallel, respectively) and unrestrained MD refined models are green (light and dark blue as the first and second parallel, respectively). The left column shows 10 representative models from the second halves of the trajectories, while the centers of the most populated clusters are shown on the right column. The most important sidechains of the binding pocket are also shown in the cluster centers. The black arrows indicate minor conformational changes in the refinements, which deviate from the reference structures.

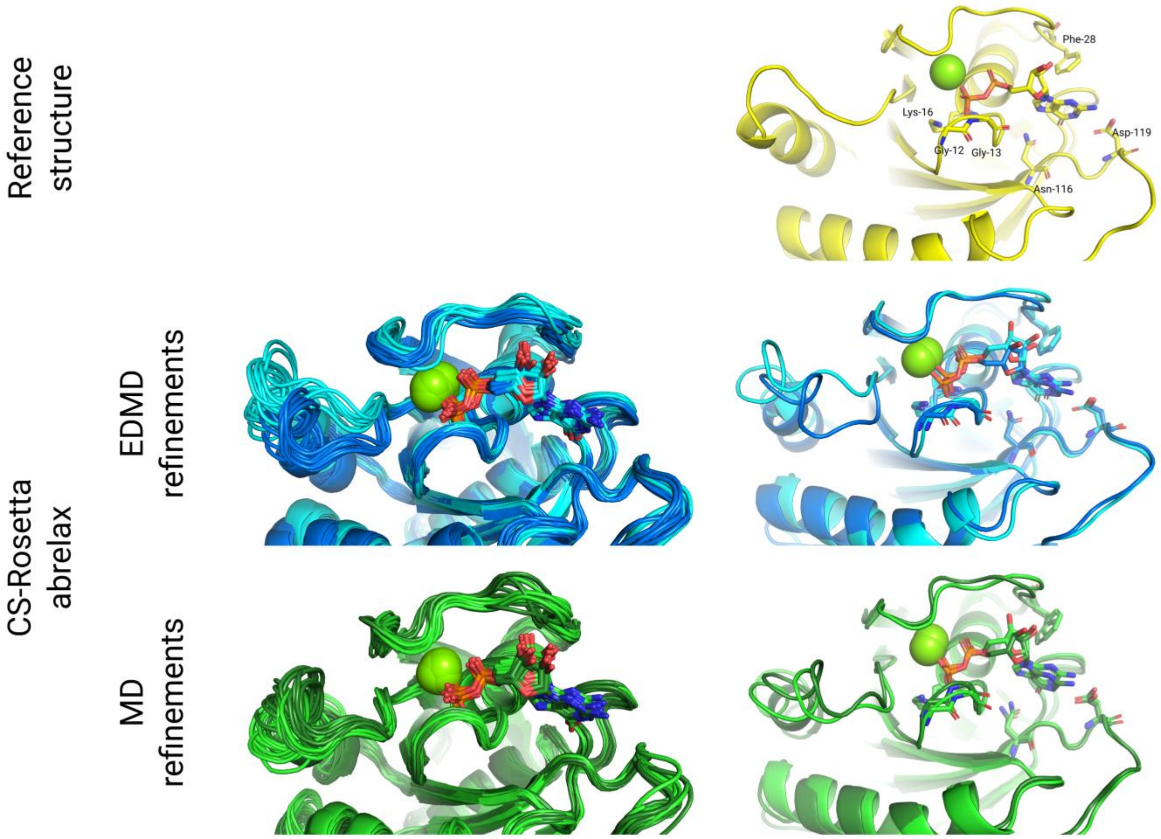

Figure S3. Overview of KRAS refinements and the GDP-binding pocket. The reference crystal structure is yellow (PDB ID: 4OBE, chain A), EDMD refined models are blue (light and dark blue as the first and second parallel, respectively) and unrestrained MD refined models are green (light and dark blue as the first and second parallel, respectively). The left column shows 10 representative models from the second halves of the trajectories, while the centers of the most populated clusters are shown on the right column. The most important sidechains of the binding pocket are also shown in the cluster centers. The greatest deviation from the crystal structure can be seen in the Switch-II region, which is allowed to sample looser conformations. This type of behavior is often seen in case of KRAS, in fact, this segment is frequently missing from the deposited crystal structures of this state[35].

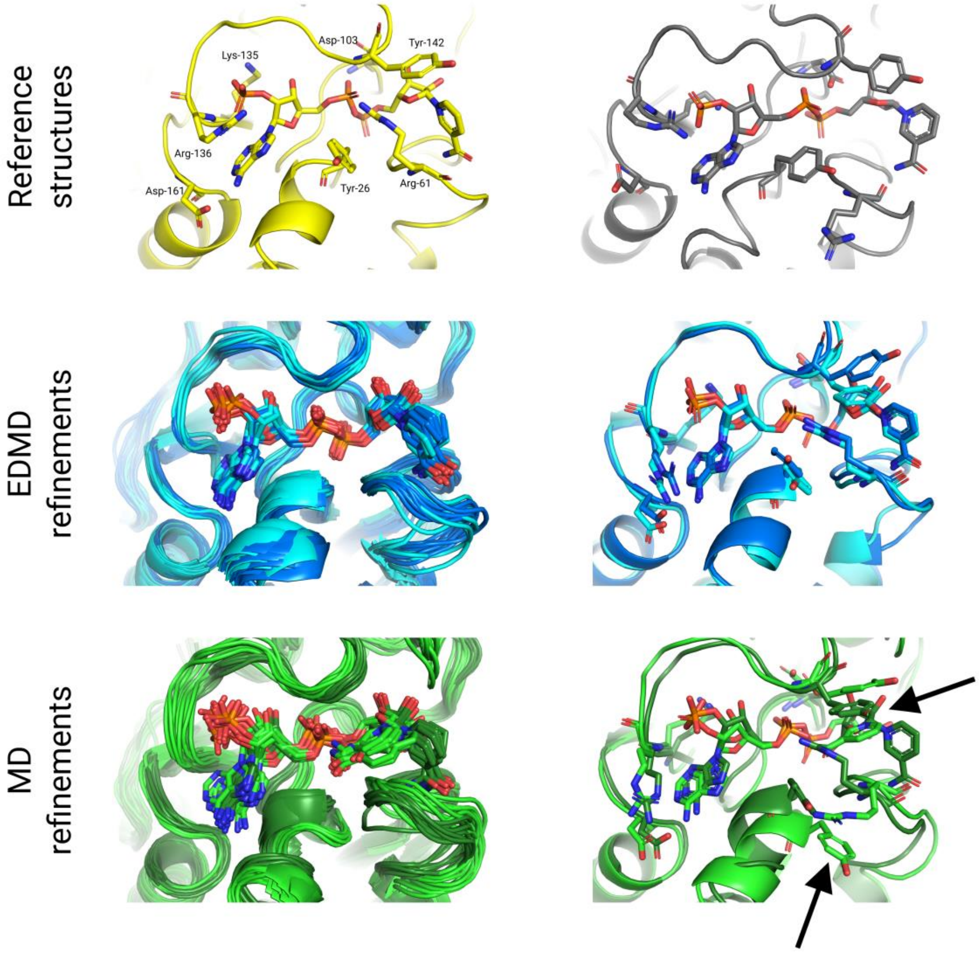

Figure S4. Overview of THdIII refinements and the NADP-binding pocket. The reference crystal structure is yellow (PDB ID: 1PNO, chain B), the reference NMR model is gray (PDB ID: 1E3T), EDMD refined models are blue (light and dark blue as the first and second parallel, respectively) and unrestrained MD refined models are green (light and dark blue as the first and second parallel, respectively). The left column shows 10 representative models from the second halves of the trajectories, while the centers of the most populated clusters are shown on the right column. The most important sidechains of the binding pocket are also shown in the cluster centers. The black arrows indicate minor conformational changes in the refinements, which deviate from the reference structures.

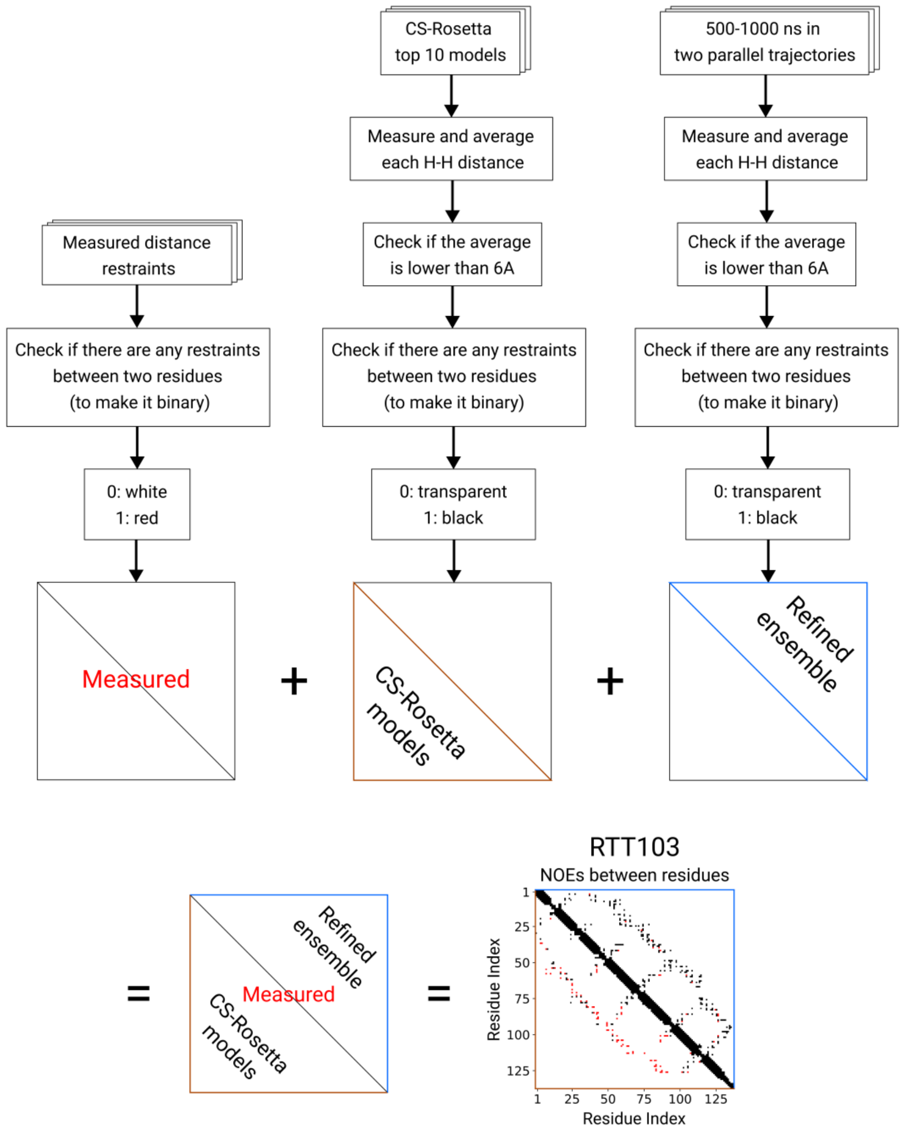

Figure S5. A block diagram showing the creation of the asymmetric binary matrices (**Figure 3B**). Three matrices were created. The first matrix is a symmetrical one, which shows if there is a distance restraint between two residues defined from the NMR NOE measurements (restraint present = red pixel, white otherwise). This matrix is the lowest layer. The second component of the figure is a half matrix on the bottom triangle, which shows whether two residue pairs are close enough on average in the best 10 CS-Rosetta models to form NOE signals (i.e., closer than 6 Å). The third component of the picture is a half matrix placed in the upper triangle. This shows whether two residues are close enough in space on average in the second part (500-1000 ns) of the two parallel simulations to form NOE signals (again, closer than 6 Å). The second and third

components form the top layer. On the top layer the pixel is black if the residues are close enough on average, and transparent, if they are not (so the lower layer can be seen). We did not take into account in either case which H atoms are closer than the threshold, just which residue they belongs to. For example: if a residue pair is closer than 6 Å in the best 10 CS-Rosetta models or in the refined ensembles, then the pixel between them is black. If they are not close enough, but there is a measured NOE between them, the pixel is red.

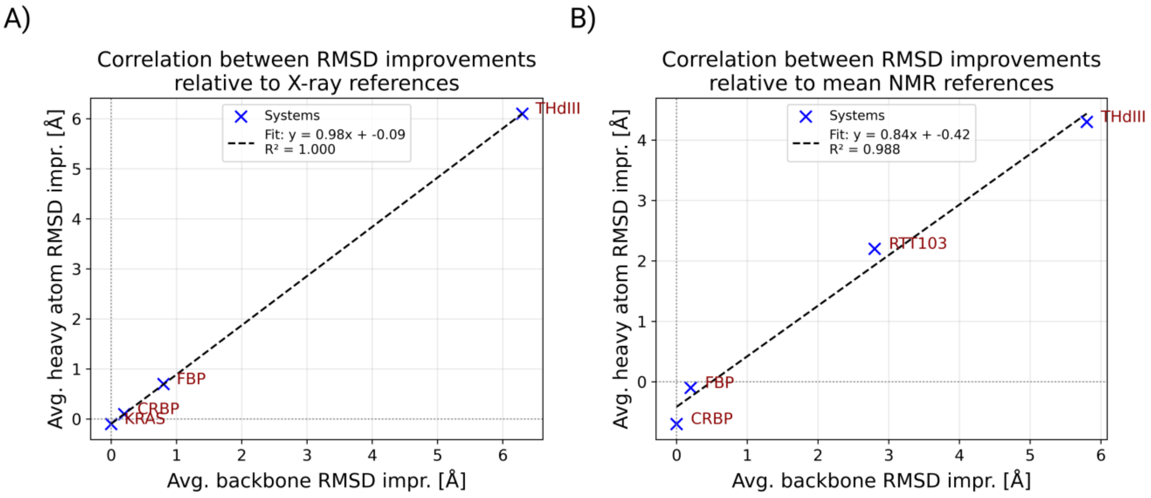

Figure S6. The linear relationship between backbone and heavy atom RMSD improvements relative to the reference crystal structures (**A**) and mean NMR structures (**B**). The PDB ID of the reference structures are listed in the **Methods** section of the Manuscript. The RMSD improvements represented here are the difference between the average RMSD in the two parallel EDMD refined trajectories (500 to 1000 ns) and average RMSD of the top 10 CS-Rosetta models, both relative to the corresponding reference structure.

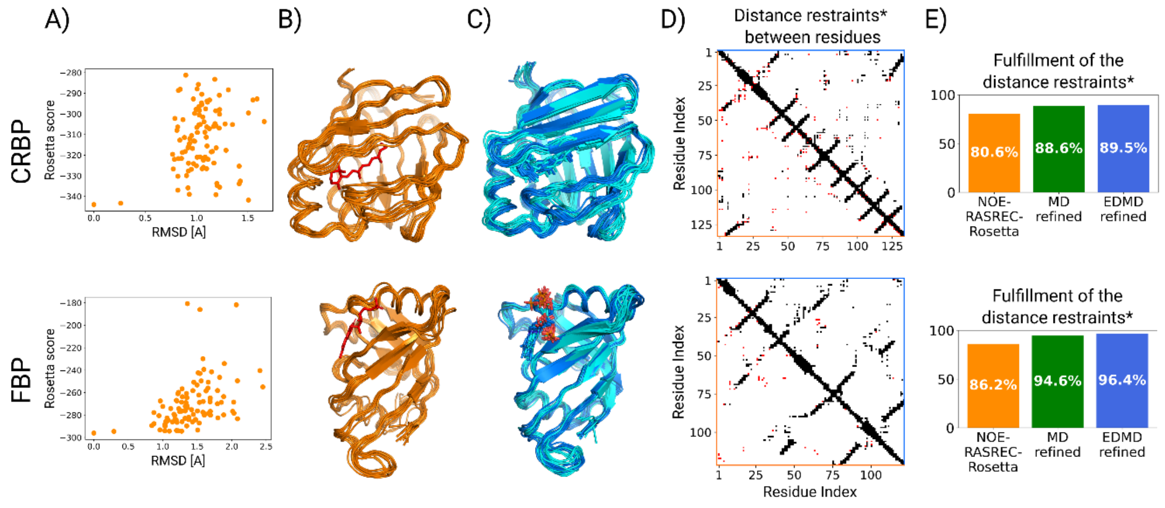

Figure S7. Summary of the NOE-RASREC-Rosetta structure determinations (orange) for CRBP and FBP and their EDMD refinements (blue) are shown. **A)** Rosetta scores versus the backbone RMSD relative to the best scoring model is shown. **B)** The 10 best scoring NOE-RASREC-Rosetta models as orange cartoon. The missing small molecules are also shown as red sticks from the reference X-ray crystal structure. **C)** 10 representative models from each parallel EDMD simulations (first parallel with light blue, second parallel with dark blue cartoons). The models also show the small molecules as sticks. **D)** The matrices show whether there are any NOE distance restraints between residue pairs. Black pixels represent that the amino acids are close enough on average in the EDMD models to form NOE signals, red points represent measured distance restraints and the lack of predicted distance restraints in EDMD refined models. **E)** Fulfillment of the distance restraints in percentages for NOE-RASREC-Rosetta models (orange), MD refined models from simulation with the original force field (green), and EDMD refined models (blue). The exact numbers for each simulation are shown in **Table S3**.

* During the calculation of the percentages only it was considered if two residues are close enough to form NOE signals, but not which protons specifically (as opposed to the NOE patterns in **Figure S7D**).

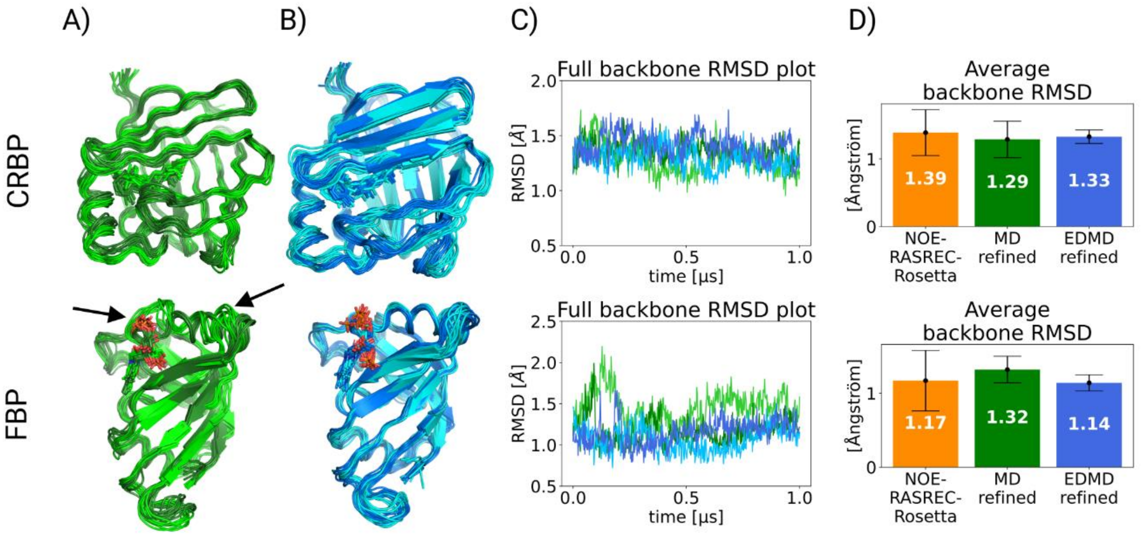

Figure S8. Results of MD refinement (using the original force field) and EDMD refinement results after the NOE-RASREC-Rosetta structure determination. **A)** The 10 models that represent the results of each of the two parallel simulations of the MD refinement (shown light and dark green, separately). The second halves of the 1000 ns simulations are considered as results. Discrepancies between parallel simulations are labeled with black arrows. **B)** The 10 models that represent the results of each of the two parallel simulations of the EDMD refinement (shown light and dark blue, separately). The second halves of the 1000 ns simulations are considered as results. **C)** Backbone RMSD of each simulation is depicted. **D)** The average backbone RMSD and its standard deviation is shown for the 10 best NOE-RASREC-Rosetta models (orange), the MD refinement (second halves of the 2 parallels, green), and the EDMD refinement (second halves of the 2 parallels, blue) relative to the X-ray crystal structure. The exact percentages for each single simulation are shown in **Table S1**.

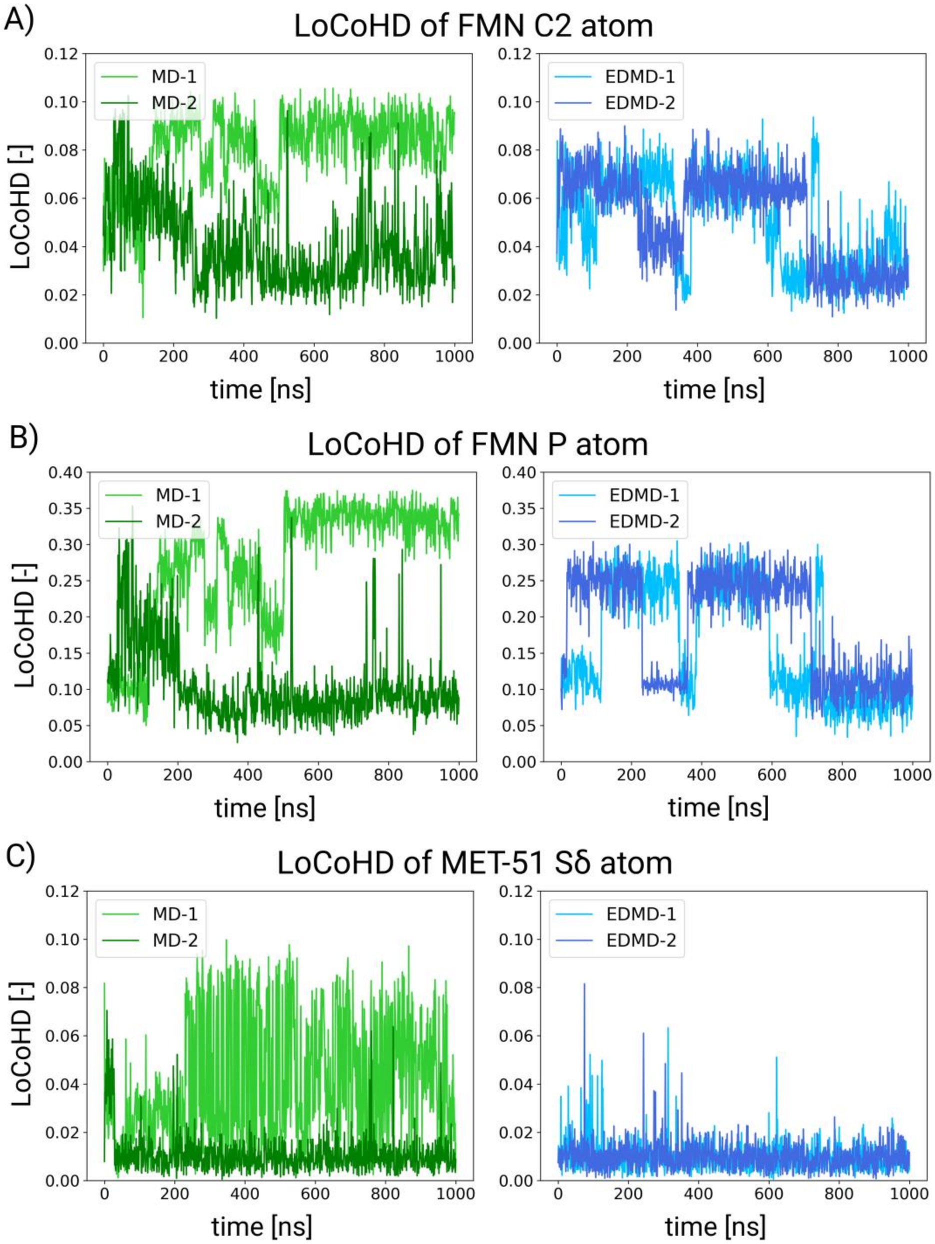

Figure S9. Local composition Hellinger distances (LoCoHD) in FBP refinement simulations based on NOE-RASREC-Rosetta calculations. LoCoHD of the environments of the FMN C2 and P atoms, and Met-51 Sδ (delta sulfur) atom were studied. As a reference the same environments of the crystal structure (PDB ID: 1FLM) were used. LoCoHD values of the two parallel MD and EDMD simulations are shown as a function of simulation time. The MD-1 trajectory shows minor conformational changes (as discussed in the **The EDMD force field is conformation specific** section of the **Results**), which is nicely represented here in the elevated LoCoHD values of the studied atoms.

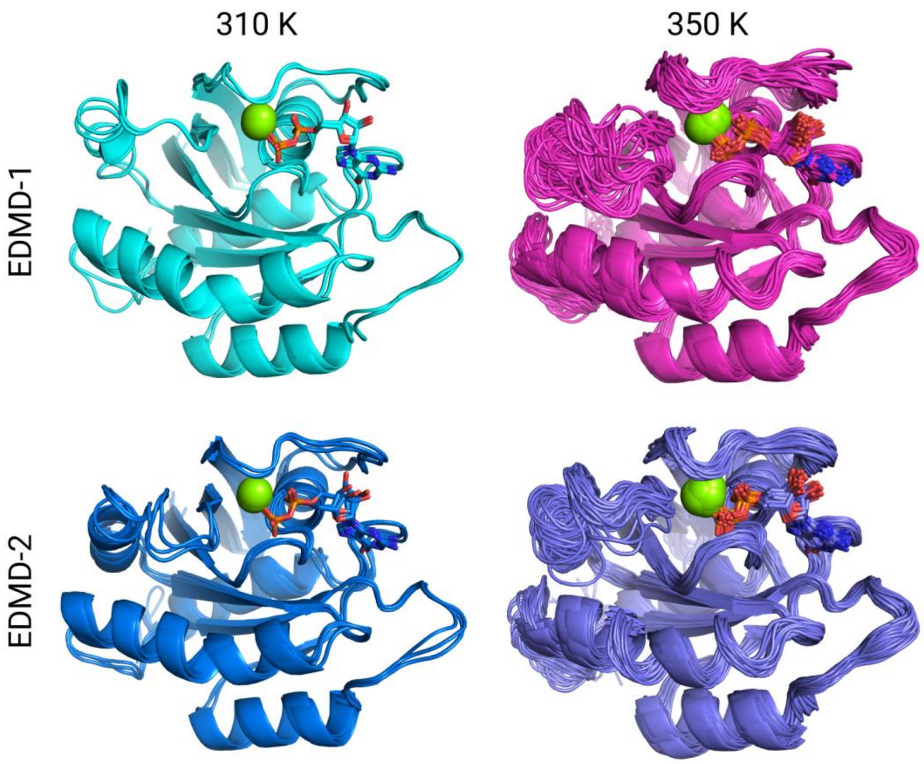

Figure S10. The temperature dependence of EDMD simulations of KRAS. The protein is shown as cartoons, GDP is depicted as sticks, and Mg^{2+} ion is represented as green spheres. Cluster centers are displayed for clusters that together account for 90% of the trajectory frames, using a clustering cutoff of 1.0 Å. 2 and 3 clusters cover 90% of the frames at 310K, which values are 40 and 33 at 350K.

Table S1. Average backbone root mean square deviation (RMSD) of each CS-Rosetta calculation (top 10 models) and each parallel refinement simulation between 500-1000 ns (both MD and EDMD) relative to the crystallographically determined reference structures (PDB ID: 5LJB for CRBP, chain A of 1FLM for FBP, chain A of 4OBE for KRAS, and chain B of 1PNO for THdIII). The RMSD values are in Ångström.

* The labelled data corresponds to the less successful MD simulations, where minor conformational changes occur, which goes hand in hand with higher RMSD.

** Average backbone RMSD of the reference NMR models relative to the crystallographically determined reference structures. In the reference NMR structures there are 25 models for CRBP (PDB ID: 1MX8), 20 for FBP (PDB ID: 1AXJ), 10 for RTT103 (PDB ID: 5WOZ), and 1 for THdIII (PDB ID: 1E3T).

		CRBP	FBP	KRAS	RTT103	THdIII
	NMR ref**	1.6 ± 0.1	2.1 ± 0.1	-	-	3.7
Abrelax	CS-Rosetta	1.7 ± 0.3	1.7 ± 0.4	1.5 ± 0.2	-	7.4 ± 4.0
	MD-1	1.6 ± 0.1	1.2 ± 0.2	1.7 ± 0.1	-	1.8 ± 0.1 *
	MD-2	2.0 ± 0.2 *	1.2 ± 0.2	1.8 ± 0.1	-	1.1 ± 0.1
	EDMD-1	1.5 ± 0.1	1.0 ± 0.1	1.3 ± 0.1	-	1.2 ± 0.1
	EDMD-2	1.5 ± 0.1	0.9 ± 0.1	1.6 ± 0.1	-	1.1 ± 0.1
NOE-RASREC	CS-Rosetta	1.4 ± 0.2	1.2 ± 0.2	-	-	-
	MD-1	1.2 ± 0.1	1.5 ± 0.1 *			
	MD-2	1.3 ± 0.1	1.2 ± 0.1			
	EDMD-1	1.3 ± 0.1	1.1 ± 0.2			
	EDMD-2	1.4 ± 0.1	1.2 ± 0.1			

Table S2. Average backbone RMSD of each CS-Rosetta calculation (top 10 models) and each parallel refinement simulation between 500-1000 ns (both MD and EDMD) relative to the mean NMR reference structures. The RMSD values are in Ångström.

* The labelled data corresponds to the less successful MD simulations, where minor conformational changes occur, which goes hand in hand with higher RMSD.

** Average backbone RMSD of the reference NMR structures relative to the mean NMR reference structures. In the reference NMR structures there are 25 models for CRBP (PDB ID: 1MX8), 20 for FBP (PDB ID: 1AXJ), 10 for RTT103 (PDB ID: 5WOZ), and 1 for THdIII (PDB ID: 1E3T).

		CRBP	FBP	KRAS	RTT103	THdIII
	NMR ref**	0.9 ± 0.1	0.5 ± 0.1	-	0.7 ± 0.2	0
Abrelax	CS-Rosetta	1.7 ± 0.3	2.4 ± 0.2	-	5.3 ± 2.4	8.4 ± 3.2
	MD-1	1.8 ± 0.1	2.0 ± 0.3		2.9 ± 0.1	3.0 ± 0.1 *
	MD-2	2.3 ± 0.2 *	2.1 ± 0.2		2.3 ± 0.1	2.6 ± 0.1
	EDMD-1	1.7 ± 0.1	2.2 ± 0.2		2.5 ± 0.1	2.6 ± 0.1
	EDMD-2	1.7 ± 0.1	2.1 ± 0.2		2.6 ± 0.2	2.6 ± 0.1
NOE-RASREC	CS-Rosetta	1.3 ± 0.1	1.9 ± 0.2	-	-	-
	MD-1	1.6 ± 0.1	2.5 ± 0.2 *			
	MD-2	1.7 ± 0.1	2.1 ± 0.2			
	EDMD-1	1.6 ± 0.1	2.1 ± 0.2			
	EDMD-2	1.7 ± 0.1	2.2 ± 0.2			

Table S3. Fulfillments of the NOE distance restraints in the whole protein for each CS-Rosetta calculation (top 10 models) and each parallel refinement simulation between 500-1000 ns (both MD and EDMD). During the calculation of the percentages only it was taken into account if two residues are close enough to form NOE signals, but not which protons specifically.

* The labelled data corresponds to the less successful MD simulations, where minor conformational changes occur, which goes hand in hand with lower fulfillment of distance restraints.

		CRBP	FBP	KRAS	RTT103	THdIII
Abrelax	CS-Rosetta	77.5 %	82.0 %	81.3 %	23.1 %	45.5 %
	MD-1	86.5 %	96.1 %	92.8 %	77.8 %	63.0 % *
	MD-2	84.8 % *	96.1 %	91.1 %	88.0 %	63.9 %
	EDMD-1	88.1 %	96.7 %	92.2 %	82.9 %	63.7 %
	EDMD-2	87.9 %	97.0 %	91.6 %	88.0 %	64.4 %
NOE-RASREC	CS-Rosetta	80.6 %	86.2 %	-	-	-
	MD-1	89.2 %	93.4 % *			
	MD-2	89.4 %	96.7 %			
	EDMD-1	90.3 %	96.7 %			
	EDMD-2	89.7 %	97.0 %			

Table S4. Intermolecular heavy atom distances in THdIII in the reference structures and refined models based on the CS-Rosetta calculations. The values are in Ångström.

* The labelled data corresponds to the less successful MD simulations, where minor conformational changes occur, which goes hand in hand with higher atom-atom distances relative to the reference structures and the lack of some intermolecular interactions.

	X-ray ref	NMR ref	MD-1 *	MD-2	EDMD-1	EDMD-2
Tyr-26 CD1 & NADP adenosyl C8A	6.5	4.5	10.1 ± 0.8	4.7 ± 0.5	4.7 ± 0.6	4.6 ± 0.6
Tyr-26 CD2 & NADP adenosyl C8A	4.6	5.2	8.4 ± 0.8	6.6 ± 0.4	6.7 ± 0.5	6.5 ± 0.5
Tyr-26 CE1 & NADP adenosyl C8A	6.5	5.3	10.8 ± 0.9	5.4 ± 0.6	5.4 ± 0.7	5.3 ± 0.7
Tyr-26 CE2 & NADP adenosyl C8A	4.7	5.9	9.2 ± 0.9	7.1 ± 0.5	7.2 ± 0.6	7.0 ± 0.6
Asp-103 OD1 & NADP nicotinamide ribose O3D	4.6	2.5	9.1 ± 0.3	4.5 ± 0.1	2.6 ± 0.1	2.6 ± 0.1
Asp-103 OD2 & NADP nicotinamide ribose O3D	2.6	4.1	8.3 ± 0.4	2.6 ± 0.1	4.6 ± 0.1	4.5 ± 0.1

Table S5. Intermolecular heavy atom distances in FBP in the reference structures and in the refined models based on the NOE-RASREC-Rosetta calculations. The values are in Ångström.

* The labelled data corresponds to the less successful MD simulations, where minor conformational changes occur, which goes hand in hand with higher atom-atom distances relative to the reference structures and the lack of some intermolecular interactions.

	X-ray ref	NMR ref	MD-1 *	MD-2	EDMD-1	EDMD-2
Met-51 N & FMN ribityl O2’	6.7	3.9 ± 0.2	11.1 ± 1.3	6.5 ± 0.3	6.4 ± 0.5	6.4 ± 0.6
Met-51 N & FMN ribityl O4’	2.8	6.8 ± 0.1	11.4 ± 1.1	3.1 ± 0.4	3.8 ± 1.2	4.4 ± 1.5

Table S6. The average solvent-accessible surface area (SASA) of each CS-Rosetta calculation (top 10 models) and each parallel refinement simulation between 500-1000 ns (both MD and EDMD). The SASA values are calculated for the proteins only and are in nm^2. The crystallographically determined reference structures are PDB ID: 5LJB for CRBP, chain A of 1FLM for FBP, chain A of 4OBE for KRAS, and chain B of 1PNO for THdIII. In the reference NMR structures there are 25 models for CRBP (PDB ID: 1MX8), 20 for FBP (PDB ID: 1AXJ), 10 for RTT103 (PDB ID: 5WOZ), and 1 for THdIII (PDB ID: 1E3T).

* The labelled data corresponds to the less successful MD simulations, where minor conformational changes occur.

		CRBP	FBP	KRAS	RTT103	THdIII
	X-ray ref	74.4	66.9	80.0	-	76.2
	NMR ref	80.5 ± 2.2	60.3 ± 1.7	-	79.0 ± 1.8	77.2
Abrelax	CS-Rosetta	75.1 ± 2.5	69.2 ± 1.9	80.2 ± 2.6	85.1 ± 3.1	84.9 ± 5.6
	MD-1	77.1 ± 1.4	69.2 ± 1.3	86.0 ± 1.4	79.1 ± 1.7	83.8 ± 1.6 *
	MD-2	76.2 ± 1.7 *	70.2 ± 1.5	86.3 ± 1.6	80.0 ± 1.6	83.1 ± 1.4
	EDMD-1	76.3 ± 1.6	69.2 ± 1.4	89.6 ± 1.5	80.2 ± 1.9	83.9 ± 1.5
	EDMD-2	77.2 ± 1.4	68.7 ± 1.2	86.9 ± 1.5	82.5 ± 1.9	84.0 ± 1.4
NOE-RASREC	CS-Rosetta	71.6 ± 2.9	66.4 ± 1.1	-	-	-
	MD-1	76.8 ± 1.4	72.0 ± 1.4 *			
	MD-2	77.0 ± 1.4	68.4 ± 1.6			
	EDMD-1	76.2 ± 1.3	69.2 ± 1.4			
	EDMD-2	76.7 ± 1.5	68.7 ± 1.3			
